# Cell-compatible isotonic freezing media enabled by thermo-responsive osmolyte-adsorption/exclusion polymer matrices

**DOI:** 10.1038/s42004-023-01061-7

**Published:** 2023-11-29

**Authors:** Yui Kato, Yuya Matsuda, Takuya Uto, Daisuke Tanaka, Kojiro Ishibashi, Takeru Ishizaki, Akio Ohta, Akiko Kobayashi, Masaharu Hazawa, Richard W. Wong, Kazuaki Ninomiya, Kenji Takahashi, Eishu Hirata, Kosuke Kuroda

**Affiliations:** 1 Faculty of Biological Science and Technology, Institute of Science and Engineering, Kanazawa University, Kakuma-machi, Kanazawa 920-1192 Japan; 2 University of Miyazaki, Faculty of Engineering, Nishi 1-1 Gakuen Kibanadai, Miyazaki, 889-2192 Japan; 3 Genetic Resource Center, National Agriculture and Food Research Organization, Kannondai, Tsukuba 305-8602 Japan; 4 Cancer Research Institute, Kanazawa University, Kakuma-machi, Kanazawa 920-1192 Japan; 5 Faculty of Material Science and Technology, Institute of Science and Engineering, Kanazawa University, Kakuma-machi, Kanazawa 920-1192 Japan; 6 Cell-Bionomics Research Unit, Institute for Frontier Science Initiative & WPI-Nano Life Science Institute, Kanazawa University, Kanazawa, Kanazawa, Ishikawa 920-1192 Japan; 7 Institute for Frontier Science Initiative, Kanazawa University, Kakuma-machi, Kanazawa 920-1192 Japan; 8 Nano Life Science Institute of Kanazawa University, Kakuma-machi, Kanazawa 920-1192 Japan; 9 NanoMaterials Research Institute, Kanazawa University, Kanazawa, Japan

**Keywords:** Biomaterials - cells, Biopolymers, Biomaterials - cells

## Abstract

During the long-term storage of cells, it is necessary to inhibit ice crystal formation by adding cryoprotectants. Non-cell-permeable cryoprotectants have high osmotic pressure which dehydrates cells, indirectly suppressing intracellular ice crystal formation. However, the high osmotic pressure and dehydration often damage cells. Emerging polymer-type non-cell-permeable cryoprotectants form matrices surrounding cells. These matrices inhibit the influx of extracellular ice nuclei that trigger intracellular ice crystal formation. However, these polymer-type cryoprotectants also require high osmotic pressure to exert an effective cryoprotecting effect. In this study, we designed a poly(zwitterion) (polyZI) that forms firm matrices around cells based on their high affinity to cell membranes. The polyZI successfully cryopreserved freeze-vulnerable cells under isotonic conditions. These matrices also controlled osmotic pressure by adsorbing and desorbing NaCl depending on the temperature, which is a suitable feature for isotonic cryopreservation. Although cell proliferation was delayed by the cellular matrices, washing with a sucrose solution improved proliferation.

## Abstract (web summary)

Non-cell-permeable cryoprotectants exhibit cryopreserving effects by preventing intracellular ice crystal formation, but these can damage cells due to high osmotic pressure and dehydration. Here, the authors developed a poly(zwitterion) isotonic cryoprotectant that forms a firm matrix around cells that prevents the influx of ice crystal nuclei without the need for high osmotic pressure and dehydration.

## Introduction

The cryopreservation of cells is an essential part of modern medicine^1^. Between 2010 and 2014, approximately 34,753 cases of egg cryopreservation for fertility treatment were reported in 17 European countries^2^. When cells are cryopreserved, ice crystals tend to form intra- and extracellularly, causing physical damage^3^. Therefore, to suppress ice-crystal formation, cryoprotectants are added. At present, two types of cryoprotectants are available: cell-permeable and non-cell-permeable. Dimethyl sulfoxide (DMSO) is a common cell-permeable cryoprotectant^4,5^ that inhibits ice-crystal formation by interacting with intra- and extracellular water molecules^6^. However, DMSO is known to permeate cells and disrupt intracellular functions, and in severe cases, induces cell apoptosis and mis-differentiation^7–10^. Sucrose and trehalose are typical non-cell-permeating cryoprotectants, and are considered less harmful as they do not permeate the cells^11^. Although non-cell-permeating cryoprotectants suppress extracellular ice crystal formation by interacting with extracellular water molecules, they are disadvantaged in that they cannot directly suppress intracellular ice crystal formation. Therefore, they act by dehydrating cells and thus indirectly suppress intracellular ice crystal formation by hypertonicity (2–3 times that of the isotonic pressure). The hypertonicity induces rapid cell dehydration, leading to significant cell damage^12^. Therefore, cryopreservation with non-cell-permeating cryoprotectants at isotonic conditions is ideal when considering cell health.

Here, we focused on freeze-concentration during the cryopreservation process. In the case of slow freezing, the water content of the freezing media is concentrated approximately 10–20 times at subzero temperatures because ice crystals exclude the solute molecules when forming and growing. Even if solutions are isotonic at room temperature, sufficient osmotic pressure for dehydration can theoretically be obtained at subzero temperatures. However, dehydration in isotonic solutions through freeze-concentration practically does not work without any breakthrough because the inside of the cells freezes before freeze-concentration has occurred.

The mechanism of intracellular freezing involves the inflow of extracellular ice nuclei^13^. A polyampholyte, carboxylated ɛ-poly-L-lysine, which was developed by Matsumura et al., has been reported to form matrices around the cell membrane and partly prevent the influx of extracellular ice nuclei^14,15^. Carboxylated ɛ-poly-L-lysine exhibits cryoprotective effects in isotonic solutions; however, solutions with an osmotic pressure of 1.3 to 2.3 times that of isotonic solutions are optimal. This suggests that the polymer matrices do not completely prevent the influx of ice nuclei. In other words, polymers that form firmer matrices are expected to be efficient in isotonic conditions.

**Fig. 1:**
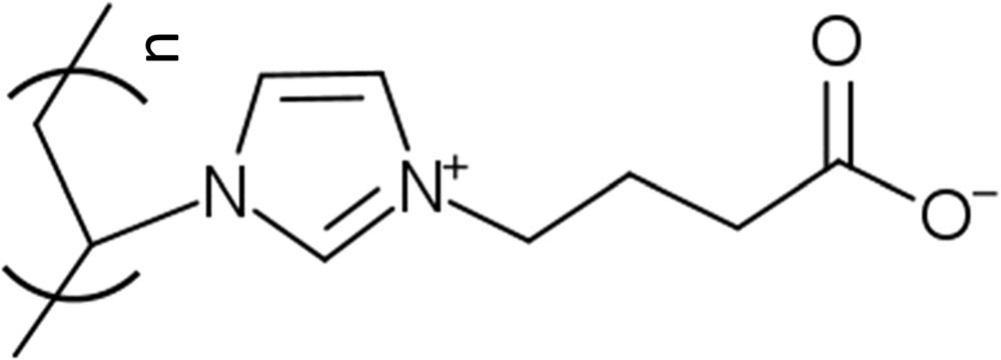
Structure of the poly(zwitterion) (PolyZI) used in this study. PolyZI was synthesized by radical polymerization of zwitterion (Supplementary Fig. 1).

In this study, we designed a zwitterion (ZI) polymer (polyZI, Fig. 1) that is expected to interact more strongly with the cell membrane than carboxylated ɛ-poly-L-lysine. We previously proposed a low-molecular ZI as a novel cryoprotectant^16,17^. ZIs are a family of ionic liquids in which anions and cations are covalently bound^18,19^. Artificial ionic liquids generally have high cytotoxicity^20–23^; however, ZIs are known to have low toxicity^16,17,19,24,25^. ZIs strongly interact with water molecules because of their charge^19^ and thus, suppress ice crystal formation^16,17^. Based on these properties, we hypothesize that ZIs would be effective cryoprotectants. Furthermore, the anions of ZIs strongly interact with the ammonium cation of the cell-membrane head group due to its charge^17^. Therefore, polyZIs are expected to be effective cryoprotectants and from firmer matrices than carboxylated ɛ-poly-L-lysine. The firm polyZI matrices reduce the optimized osmotic pressure for cryopreservation, generating an isotonic condition, which is the most biocompatible condition.

## Results and discussion

### Isotonic polyZI solutions as freezing media

PolyZIs were synthesized by introducing a vinyl group into an imidazolium/carboxylate-type ZI and an imidazolium/sulfonate-type ZI, which have demonstrated good performance as low-molecular ZIs^17^. The carboxylate-type polyZI was readily soluble in ultrapure water, while the sulfonate-type polyZI was not (Supplementary Fig. 1). Therefore, we used the carboxylate-type polyZI as the cryoprotectant in all subsequent experiments.

Two types of cells [mouse normal fibroblasts (mNF) and human kidney cells (BOSC)] were cryopreserved using the polyZI. Low-molecular ZIs generally result in moderate and low viability after the cryopreservation of mNF and BOSC, respectively^17^. For comparison, the imidazolium/carboxylate-type ZI monomer (the structure: Supplementary Fig. 1), which is the starting material of polyZI, was used as the low-molecular ZI. The relative number of living cells after freeze/thaw was used as an indicator in this study; the living cell numbers obtained after using the polyZI solutions were normalized to that obtained after using a commercial freezing medium (Culture Sure Freezing Medium, containing DMSO). The relative number of living cells after the freeze/thaw was confirmed to be low when a 10% (w/v) ZI monomer aqueous solution (aq.) was used (mNF: 0.46, BOSC: 0.10). Moreover, the relative number of living cells was unexpectedly low when a 10% (w/v) polyZI aq. was used (mNF: 0.02, BOSC: 0.2; Supplementary Fig. 2). This was caused by the low osmotic pressure of the polyZI aq. Osmotic pressure is determined by the molarity of solutes. Due to the low molarity of the polyZI aq., which was caused by the high-molecular-weight, it was speculated that the osmotic pressure was lower than the isotonic pressure and the cells became swollen. The osmotic pressure of the 10% (w/v) polyZI aq. was only 5 mOsm, which is extremely hypotonic (cf., culture medium: ~300 mOsm. 10% (w/v) ZI monomer aq.: 860 mOsm; Supplementary Table 1).

**Fig. 2:**
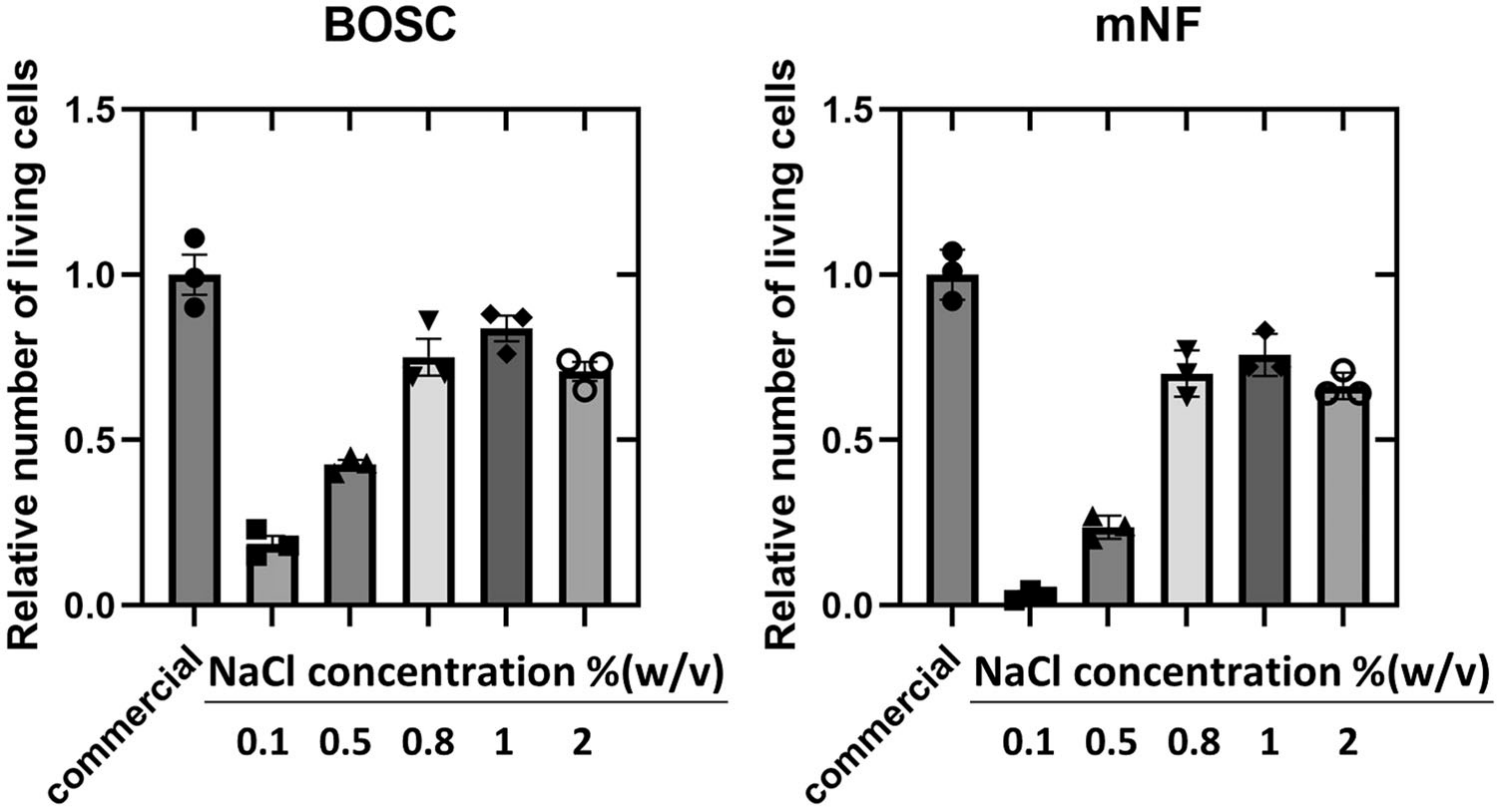
Comparison of viability after cryopreservation using polyZI with varying concentrations of NaCl. Relative number of living human kidney cells (BOSC) and mouse normal fibroblasts (mNF) after cryopreservation in 10% (w/v) polyZI aq. with the indicated NaCl concentration (*n* = 3, biological triplicates, one-way ANOVA). A commercially available freezing media (shown as “commercial”, Culture Sure freezing medium from Fujifilm Wako Pure Chemical Corporation; containing dimethyl sulfoxide (DMSO) and albumin) was used as a positive control.

NaCl was added to adjust the osmotic pressure to isotonic. As expected, the addition of 0.1% to 2.0% (w/v) NaCl improved the viability of the cells (Fig. 2). In particular, the highest relative number of living cells was observed at 1.0% (w/v) NaCl, which is approximately isotonic with saline and the medium (cf., 10% (w/v) polyZI/1.0% (w/v) NaCl aq.: 289 mOsm; phosphate-buffered saline (PBS): 280 mOsm; culture medium: 328 mOsm). Thus, we discovered that the optimal condition for a freezing medium would be isotonic osmolarity.

It has been reported that the polyZI, especially a carboxylate-type polyZI, exhibits poor cryopreservation performance^26^. In the present study, we could not conclude that the difference between the previously reported PolyZI and the polyZI used in this study was significant. Nevertheless, it has been shown that the polyZI structure is a necessary condition of good cryoprotectants, but not sufficient.

To confirm that the isotonic polyZI/1.0% (w/v) NaCl aq. was not toxic to cells, the dead cell ratio was measured after a 60 min immersion in the solution at room temperature (Supplementary Fig. 3). We found that the dead cell ratio in the polyZI/1.0% (w/v) NaCl aq. was comparable to that in the medium. Specifically, the dead cell ratio in the polyZI/2.0% (w/v) NaCl aq. was lower than that in 2.0% (w/v) NaCl aq., indicating that osmotic shock was assumed to be alleviated, as carboxylated ɛ-poly-L-lysine has demonstrated previously^14^.

### Behaviour of polyZI in NaCl aqueous solutions and its relevance to the cryoprotecting effect

**Fig. 3:**
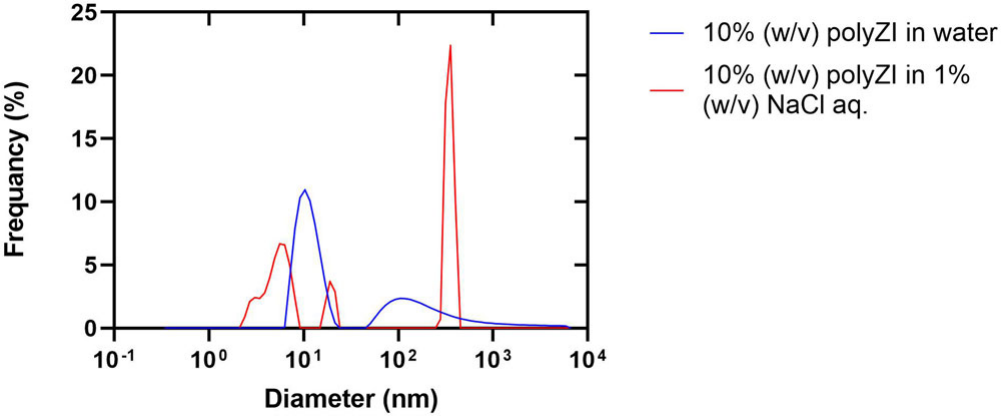
NaCl loosens polyZI aggregation. The relationship between the size of polyZI molecules and its frequency in the indicated solutions.

An understanding of the behaviour of polyZI in solutions is important for the clarification of the cryoprotecting mechanism. As mentioned previously, 10% (w/v) polyZI aq. without NaCl had an osmotic pressure of 5 mOsm, which was very low. This strongly indicates that polyZI exists in a specific state in water. For example, the osmotic pressures of the 10% (w/v) sodium polyacrylate (anionic polymer) and polyvinyl alcohol (electrically neutral polymer) aqueous solutions were 59 mOsm and 76 mOsm, respectively (Supplementary Table 2). The molecular weight of polyZI, sodium polyacrylate, and polyvinyl alcohol were 86.3, 221.4, and 69.7 kg/mol, respectively, confirming that the extremely low osmotic pressure of polyZI was not due to the molar concentration. The osmotic pressure evaluated in this study was based on the depression of vapor pressure. Considering these facts, the low osmotic pressure of polyZI aq. translates to the high remaining water activity, suggesting that the polyZI does not interact strongly with water molecules. In other words, the polyZI aggregates through intramolecular charge-mediated interactions. This suggestion is supported by the 0% unfrozen water of polyZI aq. detected using differential scanning calorimetry (DSC). More specifically, approximately 10 nm- and 100 nm-long aggregates were observed by dynamic light scattering (DLS) (Fig. 3), confirming that polyZI aggregates at the molecular level in water.

The aggregated polyZI is unlikely to exhibit cryoprotective effects; therefore, the addition of NaCl is assumed to loosen aggregation based on the electrostatic interaction. As expected, the addition of 1.0% (w/v) NaCl reduced the minimum size of polyZI from 10 nm to 3 nm, confirming that aggregation was loosened (Fig. 3). On the other hand, the addition of NaCl also increased the maximum size of polyZI from 100 nm to 120 nm. This may have been caused by the pre-treatment of the sample; the samples were passed through filters with a pore size of 3 μm, and aggregates larger than 120 nm included in the sample without NaCl were most likely removed. However, this requires more detailed consideration. These results do not conflict with those obtained by ^1^H nuclear magnetic resonance (NMR) spectroscopy. As the NaCl concentration increased, the polyZI-derived peaks became sharper. Thus, the addition of NaCl is assumed to loosen the aggregation and increase the molecular motion (Supplementary Fig. 4). This loosening effect depends on the NaCl concentration (Supplementary Fig. 5).

These results motivated us to investigate whether the loosening effect was caused specifically by NaCl. Different types of additives were added to the solution to achieve the same osmolarity as 1.0% (w/v) NaCl; sucrose, an electrically neutral sugar, and a ZI monomer and trimethylglycine (TMG), which are zwitterions. We found that these additives did not loosen aggregation, at least, although clear results were not obtained. We then hypothesized that free ions may play a role in the loosening effect; therefore, a large organic ion (an ionic liquid, 1-ethyl-3-methylimidazolium acetate) was used as an additive. However, no significant change in the aggregation state was observed; except for a slight decrease in the minimum size of the small aggregates observed at approximately 10 nm. These results indicate that free small ions, specifically NaCl, are important for the loosening of polyZI aggregation.

As mentioned above, the aggregated polyZI scarcely interacted with water (osmotic pressure: 5 mOsm, proportion of unfrozen water: 0%) when not mixed with NaCl. The interaction between polyZI and water and NaCl in NaCl aq. was investigated. We found that the addition of 10% (w/v) polyZI to 1.0% (w/v) NaCl aq. decreased the osmotic pressure from 327 mOsm to 289 mOsm, and the proportion of unfrozen water in the solution decreased from 11% to 4% (Supplementary Table 1). These results suggest an increase in water activity and indicate that the NaCl interacting with water was stripped off by the polyZI; suggesting that the polyZI/NaCl interaction is stronger than that of water/NaCl. These results suggest that NaCl was adsorbed onto the polyZI, thereby changing its structure. This strong interaction with NaCl was unique to polyZI and was not observed in the other polymers (Supplementary Table 2).

We further investigated whether the shift of the aggregation state and interaction of polyZI plays an essential role in the cryoprotecting effect. Cells were cryopreserved using solutions containing the other additives at the same osmolality as 1.0% (w/v) NaCl. The relative number of living cells was low when sucrose (neutral solute) and the ZI monomer and TMG (zwitterionic solutes) were used (Supplementary Fig. 6). When an ionic liquid (organic and free ionic solute) was used, the relative number of living BOSC cells after cryopreservation was moderate, whereas that of the mNF was low. These findings are in agreement with the above-mentioned state of loosened aggregation, and it was confirmed that aggregation loosening by adding NaCl plays an essential role in the cryoprotective effect, based not only on an osmotic pressure adjustment but also on a structure/interaction shift.

### Cryoprotective mechanism 1: inhibition of ice crystal formation

The primary cryoprotective mechanism of common cryoprotectants is associated with the inhibition of intracellular and extracellular ice crystal formation. However, the polyZI/NaCl aq. does not inhibit intracellular ice crystal formation, because it is isotonic and intracellular dehydration does not occur (Supplementary Fig. 7).

Therefore, the inhibition of extracellular ice crystal formation was considered. The proportion of antifreeze water in the polyZI/NaCl aq. was only 4% (Supplementary Table 1), which was lower than those of other low-molecular ZI solutions, DMSO solutions, and a commercial freezing medium (10–36%)^17^. This also supports the notion that the suppression of extracellular ice crystal formation is not a critical factor.

Ice recrystallisation inhibition is also a factor to consider^27,28^. However, we found that polyZI did not inhibit ice recrystallisation (Supplementary Figs. 8 and 9).

In summary, polyZI does not suppress intracellular or extracellular ice crystal formation even when freezing and thawing; thus, another factor, namely the formation of polymer matrices, is assumed to be its primary cryoprotective mechanism.

### Cryoprotective mechanism 2: polymer matrices formation

**Fig. 4:**
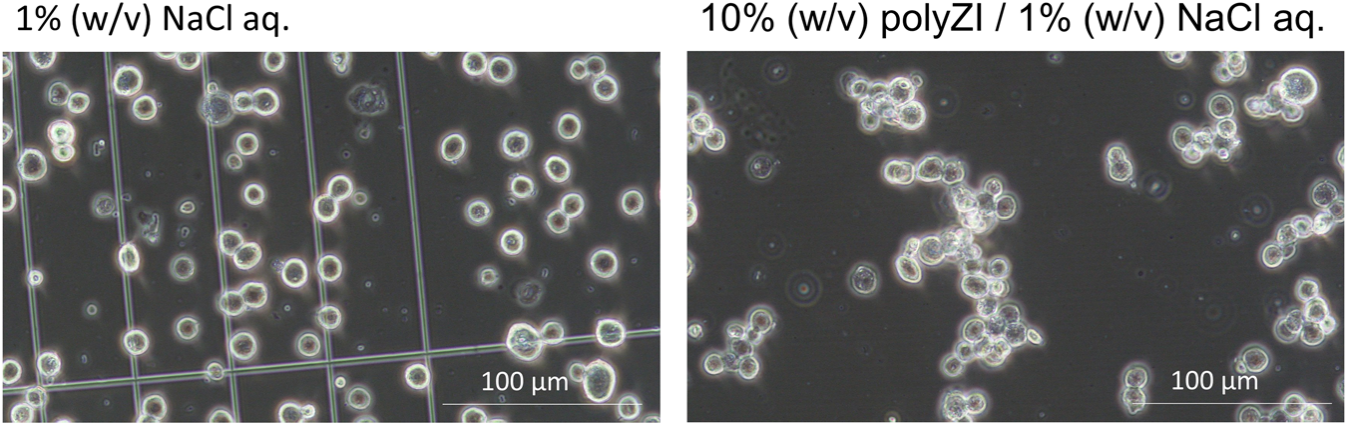
Formation of the polyZI matrices. Behaviour of BOSC cells in 1.0% (w/v) NaCl aq. and 10% (w/v) polyZI/1.0% (w/v) NaCl aq. BOSC cells immersed in the indicated solutions for 30 minutes were photographed using a microscope (ECLIPSE Ts2, Nikon Corporation).

In the present study, we sought to visualize the extracellular polyZI matrix. Initially, we attempted to synthesize polyZI with a fluorescent chromophore; however, the synthesis could not be achieved. Then we observed the cell behavior in detail and found that the cells in polyZI/NaCl aq. adhered to each other and formed aggregates (Fig. 4). This finding indirectly supports the assumption that polyZI forms matrices around the cells. It has been reported that a low-molecular ZI strongly interacts with cell membranes through an electrostatic interaction^16^. Since the polyZI does not interact strongly with water, the polyZI was expanded by the addition of NaCl and then strongly interacts with the cell membrane^29,30^ and forms matrices.

**Fig. 5:**
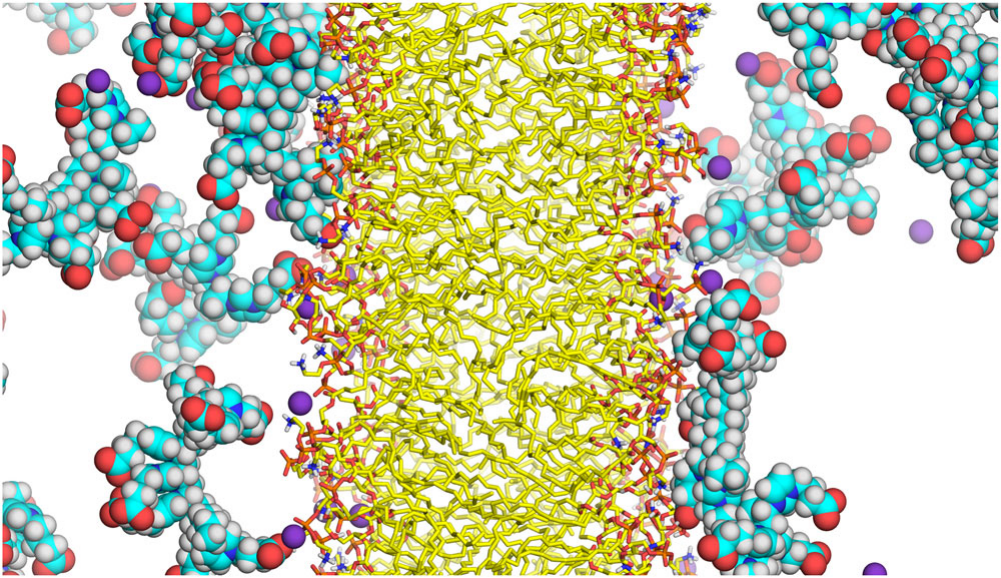
PolyZI forms a matrix around cells. Typical images of interaction between cell membrane and ZI octamer in the 10% (w/v) ZI octamer aq.

Molecular dynamics (MD) simulation supports the formation of polyZI matrix. ZI dimer and octamer were simulated. The ZI dimers and octamers partially covered the cell membrane (Fig. 5 and Supplementary Fig. 11). More specifically, we confirmed that polyZI strongly interacts with the cell membrane through ionic interactions between the amino group of dioleoylphosphatidylethanolamine (DOPE) of the cell membrane and the anion of polyZI (Supplementary Figs. 11–27, specifically Supplementary Figs. 12 and 13). This polyZI/cell membrane interaction is in equilibrium and repeats adsorption/desorption quickly. On the other hand, we observed that there are ZI octamers adsorbing onto the cell membrane for a long time. It was slightly embedded into the cell membrane by hydrophobic interactions between the oligomer backbone and 1,2-dioleoyl-sn-glycero-3-phospho-rac-(1-glycerol) (DOPG) (Supplementary Figs. 11, 20 and 21). Since the polyZI used in the experiment is 132-mer, it is assumed to adsorb onto the cell membrane for a longer period, and a large area of the cell membrane may be covered. Unfortunately, MD simulations cannot handle the long-chain polymers, like polyZI used in the experiments, due to the complexity of the initial structure determination and the computational cost.

In addition, ZI octamer aggregation was also observed in this simulation due to interaction between oligomer backbones (Supplementary Fig. 11). This agrees with the DLS results (see Fig. 3).

**Fig. 6:**
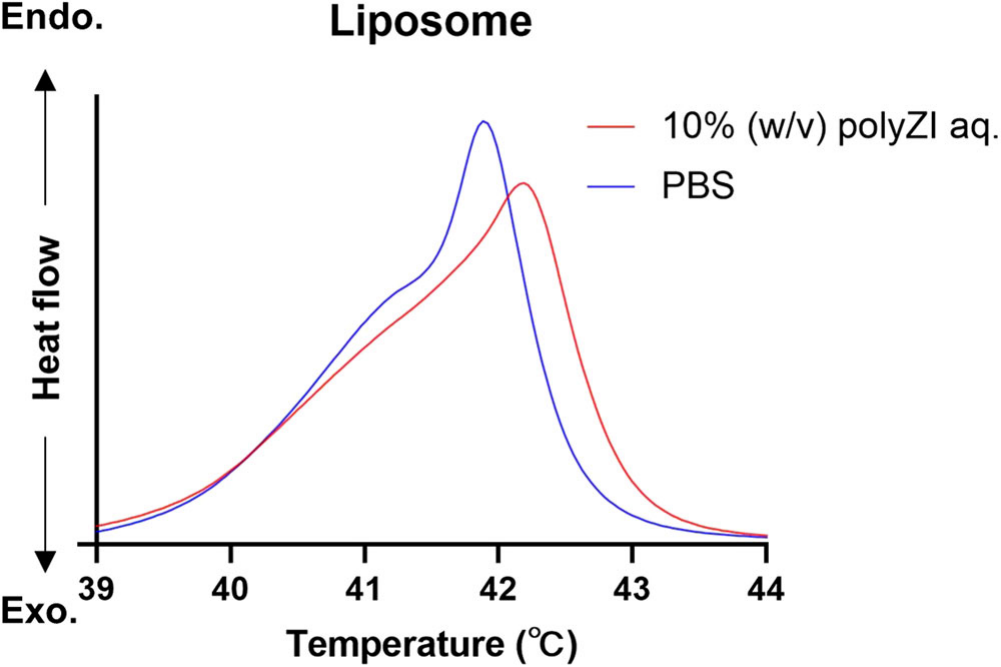
Interaction between polyZI and cell membrane. DSC chart of liposomes in PBS and 10% (w/v) polyZI solutions.

Differential scanning calorimetry (DSC) also supports the interaction between polyZI and cell membrane (Supplementary Fig. 28). The liposomes were stabilized by the addition of polyZI, which shifted the melting point of the liposomes to higher temperatures (Fig. 6). In addition, the melting point peak was broadened, meaning that the range of the melting point was widened. This is consistent with the MD simulation that polyZI partially covers the liposome rather than uniformly covers the entire cell membrane. From the data shown above, polyZI matrix forms around cells. Here we would conclude that the critical cryoprotective mechanism of polyZI/NaCl aq. is considered to be the polyZI matrix, inhibiting the influx of extracellular ice nuclei.

Matsumura et al. speculated that the matrices of carboxylated ɛ-poly-L-lysine, which is a polyampholyte, trap NaCl, thereby mitigating osmotic shock^14^. As shown in Supplementary Fig. 3, the polyZI reduced the dead cell ratio in hypertonic 2.0% (w/v) NaCl aq., which suggests that the polyZI matrix has the same function under hypertonic conditions.

**Fig. 7:**
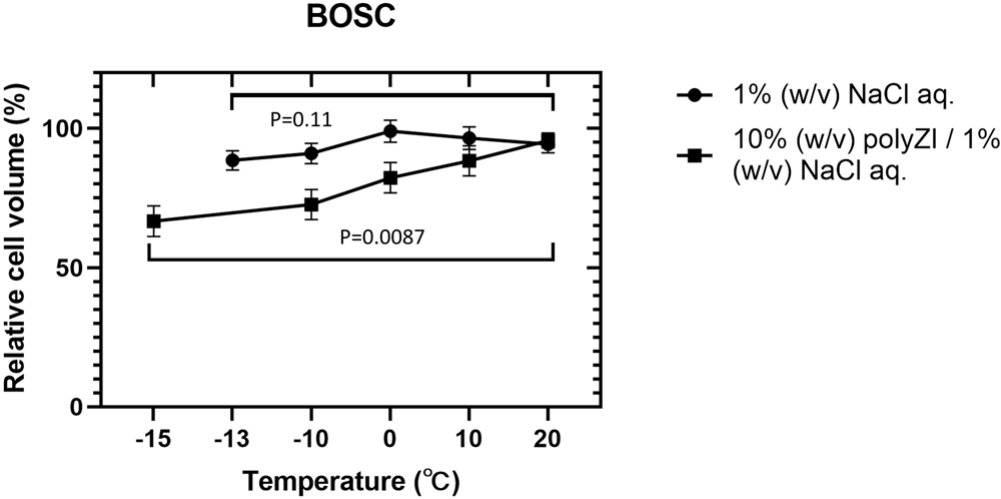
Gradual dehydration of cells in the isotonic polyZI solution. Relative BOSC cell volume in 10% (w/v) polyZI/1.0% (w/v) NaCl aq. and 1.0% (w/v) NaCl aq. at the indicated temperatures (standardized as 100% at room temperature) when cooling at −1 °C/min (*n* = 3, biological triplicates, one-way ANOVA). These cells were incubated as floating cells after trypsinization. The final cell volume was measured immediately before freezing as measurement becomes difficult after freezing.

The time course of osmotic pressure sensed by cells was estimated from the cell size (namely the volume change through cell dehydration) in the 10% (w/v) polyZI/1.0% (w/v) NaCl aq. to investigate the function of the matrix in detail. We found that the cell sizes were similar at room temperature, regardless of the addition of polyZI (Supplementary Fig. 7). When the temperature was lowered at −1 °C/min, the cells were gently dehydrated in the polyZI/NaCl aq. but not in the NaCl aq. (Fig. 7). Thus, dehydration was affected by temperature rather than soaking time (see Supplementary Fig. 7). The temperature-dependent dehydration indicates that the polyZI matrix adsorbed and locally concentrated NaCl at subzero temperatures.

In addition to preventing the influx of extracellular ice nuclei, temperature-dependent dehydration may contribute to the inhibition of intracellular ice crystal formation. Here we discuss this point, referring to cryopreservation using low-molecular ZIs. Cell dehydration is the main cryoprotective mechanism of low-molecular ZIs and the cell volume in low-molecular ZI aq. becomes 40% or less at 0 °C compared to that in a culture medium at room temperature^17^. The relative volume at 0 °C and −13 °C in polyZI/NaCl aq. was 81% and 66%, respectively, indicating that the dehydration by polyZI/NaCl alone may not be sufficient but may help the inhibition of intracellular ice crystal formation together with the polyZI matrices. More interestingly, the volume returned to its original state when the temperature increased (Supplementary Fig. 10), suggesting that adsorption/desorption was reversible and the osmotic pressure returned to normal during thawing. The cryoprotective mechanism of polyZI is not only strengthening the polymer matrix formation but also the reversible adsorption/desorption, which is partially different from that reported for polyampholytes.

The polyZI has triple functions and is suitable for cryopreservation: (1) firm polyZI matrices inhibit the inflow of extracellular ice nuclei, (2) polyZI floating in the solution absorbs NaCl to alleviate osmotic shock at room temperature, (3) the extracellular polyZI matrices concentrate NaCl to dehydrate and suppress intracellular ice crystal formation at subzero temperatures. To date, the functions (2) and (3) have not been reported in polyampholytes. More specifically, to the best of our knowledge, the function (3) has not been previously reported, while the function (2) has only been reported at different and specific conditions: after freezing and thus not at room temperature^14^. Although it is not clear whether these functions are specifically exhibited in polyZI only, and not all polyampholytes, the polyZI is assumed to adsorb NaCl more strongly than polyampholytes due to its charge. If the polyZI structure is a key factor, we speculate that the more specific key factor is: the polyZI is an aprotic polymer imparting constant charges, while carboxylated ɛ-poly-L-lysine is a protic polymer exhibiting an equilibrium of neutral/charged states. We believe that these unique functions based on the unique characteristics play a key role in the cryopreservation mechanism under isotonic conditions.

### Cell proliferation after cryopreservation in 10% (w/v) polyZI/1.0% (w/v) NaCl aq

**Fig. 8:**
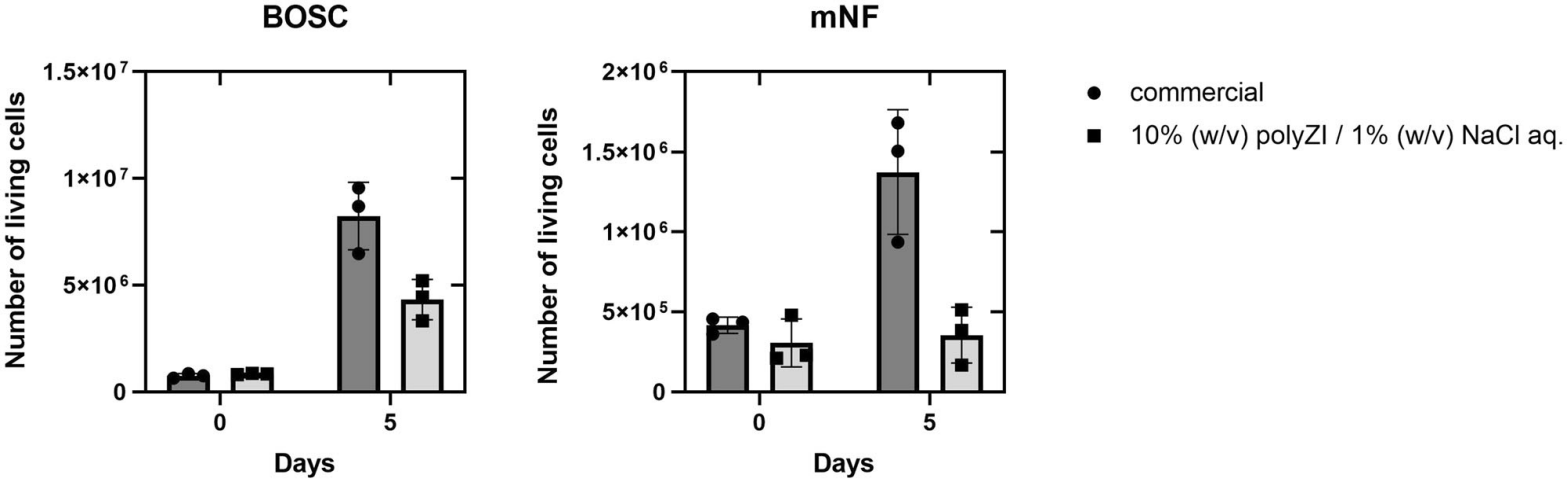
Cell growth after cryopreservation using polyZI. Number of living BOSC and mNF cells immediately and 5 days after freezing and thawing in 10% (w/v) polyZI/1.0% (w/v) NaCl aq. (*n* = 3, biological triplicates, one-way ANOVA). Commercial: Culture Sure freezing medium from Fujifilm Wako Pure Chemical Corporation.

To investigate cell proliferation after cryopreservation, the cryopreserved cells were cultured for several days, and cells were counted. Although the number of living cells in polyZI/NaCl aq. immediately after thawing was not significantly different from that in the commercial freezing medium, the proliferation of cells cryopreserved with polyZI/NaCl aq. was significantly slower (Fig. 8). We observed the cells in detail and found that cell adhesion to the dishes was less when using polyZI/NaCl aq. The polyZI matrix could play a role in the inhibition of adhesion. In this experiment, the cryoprotectant (100 μL) was washed once with a culture medium (1 mL). These results suggest that this washing process was not appropriate for the removal of the polyZI matrix.

**Fig. 9:**
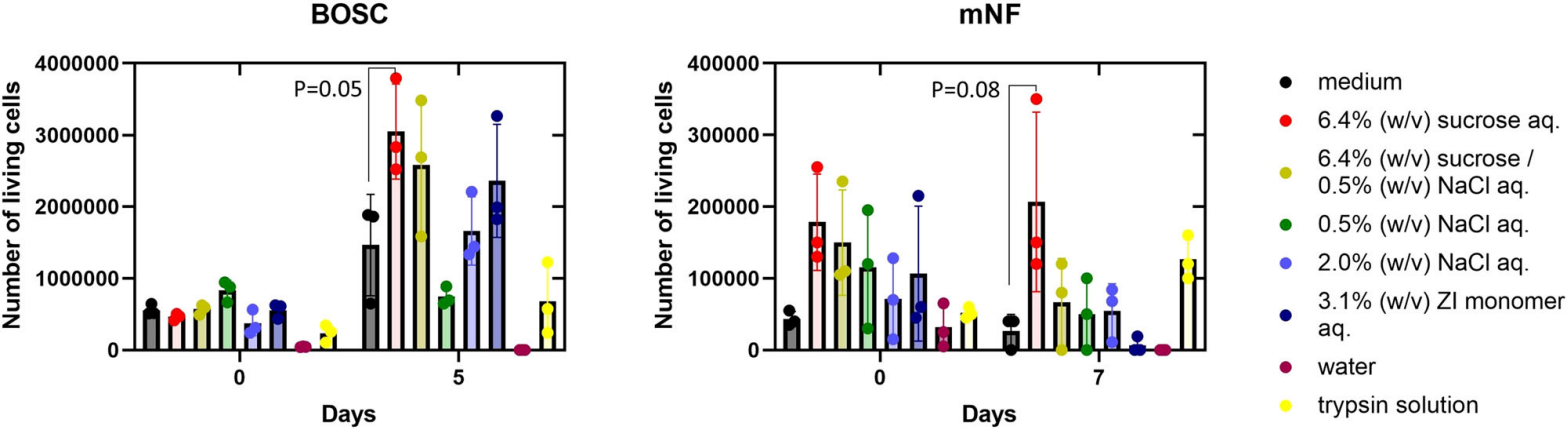
Remove polyZI after cryopreservation. Number of living BOSC cells and mNF immediately and 5–7 days after thawing and washing with the indicated solutions (*n* = 3, biological triplicates, one-way ANOVA). Commercial: Culture Sure freezing medium from Fujifilm Wako Pure Chemical Corporation. Trypsin solution: cells were added to trypsin solution (0.5 w/v% trypsin-5.3 mmol/L EDTA・4Na solution without phenol red (×10), Fujifilm Wako Pure Chemical Corporation)) that had been diluted 10 times with phosphate buffered saline (PBS) and incubated for 3 min before centrifugation.

Hence, more effective washing solvents were sought: medium, water, 0.5% (w/v) NaCl aq., 2% (w/v) NaCl aq., 3.1% (w/v) ZI monomer aq., 6.4% (w/v) sucrose aq. and 6.4% (w/v) sucrose/0.5% (w/v) NaCl aq. The 3.1% (w/v) ZI monomer aq. and the 6.4% (w/v) sucrose aq. were equimolar to the 1.0% (w/v) NaCl aq. We did not examine washing with the 1.0% (w/v) NaCl aq., because it has similar nature to the culture medium. The sucrose aq. resulted in a high living cell number after freezing, thawing, and culturing (Fig. 9). Water and the NaCl aqs. were not effective, whereas the ZI monomer aq. was only effective for BOSC. Since NaCl specifically affects the polyZI aggregation states (see Fig. 3), neutral or zwitterionic solutions are probably better than NaCl solutions. In this way, washing with sucrose solution made it easier to remove polyZI and cells proliferated, but the complete removal was not achieved at this primitive stage. Since polyZI is a new CPA, we believe that with further study we will find a way to remove it.

We also assumed that since the polyZI was attached to cells, and therefore, trypsinization may detach it from the cells. However, no positive effect was observed (see Fig. 9).

### Cryopreservation of freeze-vulnerable cells with the isotonic polyZI/NaCl aq

**Fig. 10:**
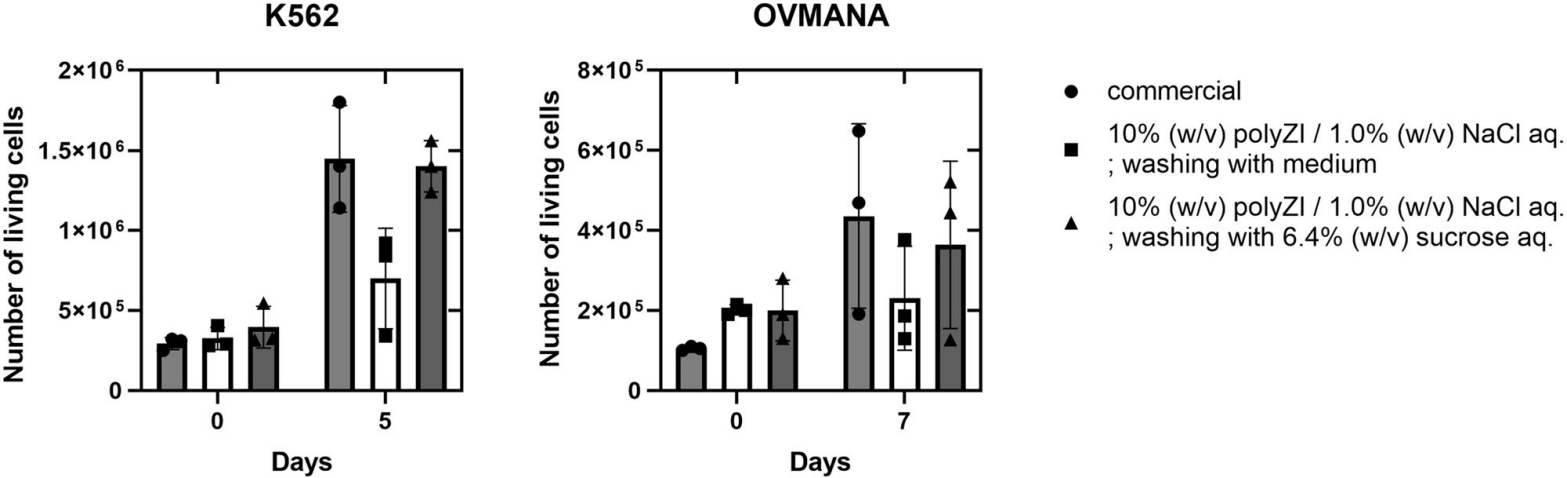
Cryopreservation of freeze-vulnerable cells with the isotonic polyZI solution. Number of living cells for K562 and OVMANA immediately and 5–7 days after cryopreserving and washing with the indicated solutions (*n* = 3, biological triplicates, two-way ANOVA). Commercial: Culture Sure freezing medium from Fujifilm Wako Pure Chemical Corporation. There is no statistical significance between the samples (*p* > 0.1).

Freeze-vulnerable cells (OVMANA and K562) were subjected to cryopreservation using the polyZI/NaCl aq. When K562 cells were cryopreserved using the polyZI/NaCl aq., the relative number of living cells immediately after thawing tends to be higher than that when using a commercial medium, suggesting the importance of isotonic cryopreservation (Fig. 10). In contrast, the number of living K562 cells cultured for 5 days after washing with 6.4% (w/v) sucrose aq. was approximately the same as that when a commercial medium was used. Similar results were obtained for OVMANA cells (Fig. 10). The polyZI/NaCl aq. was effective for freeze-vulnerable cells presumably due to avoiding the osmotic shock but the removal of the polyZI matrices is still challenging.

Because there is currently no way to completely remove the polyZI matrices, the positive effects of the isotonic freezing medium and the negative effects of adhesion inhibition may counteract each other. Nevertheless, commercial freezing mediums have been optimized for decades, and the polyZI freezing medium still has significant room; however, we do believe that it has substantial potential because the simple mixture composed of only polyZI/NaCl/water provides the same level of cryoprotective performance as the commercial medium. In this study, we designed an isotonic cryopreservation agent that does not induce osmotic stress in cells; however, further studies should be conducted to improve the washing method.

## Conclusion

We developed a 10% (w/v) polyZI/1.0% (w/v) NaCl aq. as an isotonic freezing medium. The polyZI was able to form matrices around the cell membrane and prevent influx of ice nuclei from outside the cell. Furthermore, the polyZI matrix can adsorb and desorb NaCl depending on temperature, and changes the osmotic pressure to make conditions suitable for cryopreservation. Through these mechanisms, cells were efficiently cryopreserved without suffering from strong osmotic stress. Although poor removal of the polyZI matrices prevented cell proliferation after cryopreservation, washing with sucrose aq. partially resolved this problem. Overall, we believe that the isotonic polyZI solution has the potential to enable the cryopreservation of cells which cannot currently be cryopreserved.

## Materials and methods

The ionic liquid used in the present study was purchased from Iolitech GmbH and used as received. The reagents (1-vinylimidazole, 1-bromo-2-(2-methoxyethoxy)ethane, 1,3-propenesultone. azobis(isobutyronitrile), and tetrahydrofuran) used in the synthesis of the ZI monomers and PolyZI were purchased from Tokyo Chemical Industry Co., Ltd., and used as is or recrystallized. The reagents (ethyl acetate and aluminium oxide basic) used in the synthesis of the ZI monomers were purchased from Kanto Chemical Co., Inc., and used as is. Trimethylglycine (TMG) and polyvinyl alcohol were purchased from Tokyo Chemical Industry Co., Ltd., and used as is. Sodium chloride, sodium polyacrylate, and sucrose were purchased from Nacalai Tesque, Inc. and used as is.

### Synthesis of polyZIs

The imidazolium/carboxylate- and imidazolium/sulfonate-type ZI monomers were synthesized using a previously described method with minor modifications^19,31^. The imidazolium/carboxylate ZI monomer and azobis(isobutyronitrile) were added to pure water and stirred for 16 h at 80 °C. The carboxylate-type polyZIs were obtained via dialysis with pure water and drying under reduced pressure. The imidazolium/sulfonate ZI monomer and azobis(isobutyronitrile) were dissolved in 2% NaCl aq. and stirred at room temperature for 16 h. The sulfonate-type polyZIs were obtained via reprecipitation in pure water and drying under reduced pressure. The syntheses were confirmed by ^1^H NMR (ECA400, JEOL Ltd.).

The molecular weight of the polyZI was measured by gel filtration chromatography. A TSKgel α-M column was used as the stationary phase. Pure water was used as the eluent; the flow rate was 1.0 mL/min, and the measurement temperature was 40 °C. The sample was detected using a refractive index detector (RID-10A), and the molecular weight was corrected using polyethylene oxide standards.

### Cells

Human renal cells (BOSC) were kindly provided by Professors Etsuko Kiyokawa (Kanazawa Medical University), Seiji Yano (Cancer Research Institute of Kanazawa University), and Michiyuki Matsuda (Kyoto University). Mouse normal fibroblasts (mNF) derived from C57BL/6-EGFP mice, were kindly provided by Professor Erik Sahai (The Francis-Crick Institute, UK). OVMANA human ovarian tumour cells were purchased (Japanese Collection of Research Bioresources). K562 human chronic myelogenous leukaemia cells were used^32,33^.

### Cell culture

BOSC, mNF and OVMANA cells were propagated and maintained as monolayer cultures at 37 °C in a 5% CO_2_ humidified atmosphere, using Dulbecco’s modified Eagle’s medium (DMEM, high glucose with L-glutamine and phenol red, Fujifilm Wako Pure Chemical Corporation) supplemented with 10% Foetal Bovine Serum (FBS) (Sigma-Aldrich Co., Llc.) and 1% penicillin–streptomycin solution (×100) (Fujifilm Wako Pure Chemical Corporation). K562 cells were propagated and maintained as floating cultures at 37 °C in a 5% CO_2_ humidified atmosphere, using Roswell Park Memorial Institute medium (RPMI, Nacalai Tesque, Inc.) supplemented with 10% FBS and 1% penicillin–streptomycin solution. The BOSC and mNF cells were sub-cultured every 3–4 days in the presence or absence of trypsin solution (0.5 w/v% trypsin-5.3 mmol/L EDTA・4Na solution without phenol red (×10), Fujifilm Wako Pure Chemical Corporation)). OVMANA cells were sub-cultured every 7–8 days in the presence or absence of trypsin solution (0.5 w/v% trypsin-5.3 mmol/L EDTA・4Na solution without phenol red (×10), Fujifilm Wako Pure Chemical Corporation)).

### Cryopreservation

The polyZI solutions were prepared by mixing with ultrapure water. Cells (1 × 10^6^) were harvested and centrifuged (100 × g for 5 min at room temperature). After removing the supernatant, 100 μL of freezing media was added and mixed with gentle pipetting. The samples were stored in a box (Mr. Frosty, Thermo Fisher Scientific Inc.) in a −85 °C freezer for 3–5 days. For thawing, culture media incubated at 37 °C were added to the frozen samples. The relative number of living cells was counted using a hemocytometer (Fukaekasei Corporation and Watson Corporation) after staining using trypan blue (Fujifilm Wako Pure Chemical Corporation).

$$	{{{{{\rm{Relative}}}}}}\; {{{{{\rm{number}}}}}}\; {{{{{\rm{of}}}}}}\; {{{{{\rm{living}}}}}}\; {{{{{\rm{cells}}}}}} \\ 	= \frac{{{{{{\rm{Counted}}}}}}\; {{{{{\rm{living}}}}}}\; {{{{{\rm{cell}}}}}}\; {{{{{\rm{number}}}}}}({{{{{\rm{sample}}}}}})}{{{{{{\rm{Counted}}}}}}\; {{{{{\rm{living}}}}}}\; {{{{{\rm{cell}}}}}}\; {{{{{\rm{number}}}}}}({{{{{\rm{commercial}}}}}}\; {{{{{\rm{freezing}}}}}}\; {{{{{\rm{media}}}}}})}$$

The commercial freezing media used was Culture Sure freezing medium (containing DMSO; Fujifilm Wako Pure Chemical Corporation). This is a representative commercial freezing media and is suitable for the comparison of the cryopreservation efficiencies. In this study, the relative number of living cells was adopted because the absolute number of living cells fluctuates based on biological variations even when commercial freezing media is used.

### Growth rate

After cryopreservation, all cells were seeded in 6-well plates and cultured. K562 cells were serially counted after the indicated days. BOSC, mNF and OVMANA cells were counted after trypsinization when most proliferating cells reached 80% confluence.

### Photographs of cells

Cells were soaked in polyZI solutions for 30 minutes and were photographed using a microscope (ECLIPSE Ts2, Nikon Corporation) and a camera (FLOYD, WRAYMER INC.) with a hemacytometer.

### Toxicity of cryoprotectants

After trypsinisation, BOSC (1 × 10^5^ cells) were harvested and centrifuged (100 × g for 5 min at room temperature). After removing the supernatant, 100 μL of the sample solutions were added and mixed via gentle pipetting. The cells were incubated at room temperature (air-conditioned, setting temperature was 25 °C) as cryoprotectant suspensions. After 60 min, the dead cell ratio was assessed by counting using a hemocytometer and staining with trypan blue.

$${{{{{\rm{Dead}}}}}}\; {{{{{\rm{cell}}}}}}\; {{{{{\rm{ratio}}}}}}\, \left( \% \right)=\frac{{{{{{\rm{Number}}}}}}\; {{{{{\rm{of}}}}}}\; {{{{{\rm{dead}}}}}}\; {{{{{\rm{cells}}}}}}}{{{{{{\rm{Number}}}}}}\; {{{{{\rm{of}}}}}}\; {{{{{\rm{living}}}}}}\; {{{{{\rm{cells}}}}}}+{{{{{\rm{Number}}}}}}\; {{{{{\rm{of}}}}}}\; {{{{{\rm{dead}}}}}}\; {{{{{\rm{cells}}}}}}}$$

### Cell volumes in the freezing media

BOSC (1 × 10^5^ cells) were harvested via trypsinisation and centrifuged (100 × g for 5 min at room temperature). Following the removal of the supernatant, 100 μL of the freezing media was added and mixed via gentle pipetting. The cells were then incubated at room temperature (air-conditioned, setting temperature was 25 °C) or under cooling as cryoprotectant suspensions. The images of the cells were captured using an optical inverted microscope (IX83, Olympus Corporation). The cell radii were obtained using software (ImageJ 1.52p, Wayne Rasband, National Institutes of Health, USA). The relative cell volume was calculated using the following formula:

$${{{{{\rm{Relative}}}}}}\; {{{{{\rm{cell}}}}}}\; {{{{{\rm{volume}}}}}}\;\left( \% \right)=\frac{{{{{{\rm{Cell}}}}}}\; {{{{{\rm{volume}}}}}}\; {{{{{\rm{in}}}}}}\; {{{{{\rm{sample}}}}}}\;({{{{{{\rm{\mu }}}}}}{{{{{\rm{m}}}}}}}^{3})}{{{{{{\rm{Cell}}}}}}\; {{{{{\rm{volume}}}}}}\; {{{{{\rm{in}}}}}}\; {{{{{\rm{PBS}}}}}}\;({{{{{{\rm{\mu }}}}}}{{{{{\rm{m}}}}}}}^{3})}\times 100$$

### Osmotic pressure of cryoprotectants

Osmotic pressure was measured using a vapor pressure osmometer (VAPRO 5600; Wescor, Inc., Logan, UT, USA) in a standard 10 mL chamber.

### Proportion of unfrozen water

The proportion of unfrozen water was investigated using DSC (DSC-60A plus, Shimadzu Corporation). DSC measurement was performed under the following conditions: cooling to −100 °C at a cooling rate of −1 °C/min followed by heating to 25 °C at a heating rate of 5 °C/min. The proportion of unfrozen water in the solution was estimated from the area of the melting peak at ~0 °C and calculated using the following formula:

$$	{{{{{\rm{Proportion}}}}}}\; {{{{{\rm{of}}}}}}\; {{{{{\rm{unfrozen}}}}}}\; {{{{{\rm{water}}}}}}\; {{{{{\rm{in}}}}}}\; {{{{{\rm{solution}}}}}}\;\left( \% \right) \\ 	= 100-\frac{{{{{{\rm{Melting}}}}}}\; {{{{{\rm{heat}}}}}}\; {{{{{\rm{of}}}}}}\; {{{{{\rm{the}}}}}}\; {{{{{\rm{sample}}}}}}\; {{{{{\rm{solutions}}}}}}\;[{{{{{\rm{J}}}}}}/{{{{{\rm{g}}}}}}]}{{{{{{{\rm{Melting}}}}}}\; {{{{{\rm{heat}}}}}}\; {{{{{\rm{of}}}}}}\; {{{{{\rm{water}}}}}}\;(265\;[{{{{{\rm{J}}}}}}/{{{{{\rm{g}}}}}}])} \times {{{{{\rm{water}}}}}}\; {{{{{\rm{proportion}}}}}}\; {{{{{\rm{in}}}}}}\; {{{{{\rm{the}}}}}}\; {{{{{\rm{solution}}}}}}} \times 100$$

### Ice recrystallization Inhibition

Two microlitre droplets of the polymer solutions dissolved in 20% sucrose solutions were dropped onto glass microscope coverslips. All of the samples were rapidly cooled from room temperature to −50 °C at a rate of 20 °C/min. Samples were held for 2 min at −50 °C and then warmed at a rate of 10 °C/min to −8 °C, where the samples were held for 30 min. The images of the ice crystals were captured using a microscope (ECLIPSE Ts2, Nikon Corporation) with a cooling stage (10002 L, JAPAN HIGH TECH CO., LTD.). The 5–10 largest ice crystals were selected to measure MLGS. This was then compared with the 20% sucrose solution control.

### Behaviour of polyZI detected by NMR

^1^H NMR was measured using ECA400 (external magnetic field 400 MHz) manufactured by JEOL Ltd. The 10% (w/v) polyZI was dissolved in D_2_O and measured at room temperature.

### Size of polyZI aggregation

For particle size distribution, a DLS device manufactured by HORIBA, Ltd. was used. The 1% (w/v) solution was passed through a 3 μm filter and put into a disposable cell, and the particle size was measured at a set temperature of 25 °C and at a manual time of 120 s. After measurement, the sample was analyzed with polyvariance at a threshold of 0.

### MD simulation

The cell membrane in the molecular dynamics (MD) simulation system was composed of dioleoylphosphatidylethanolamine (DOPE) and dioleoylphosphatidylglycerol (DOPG) lipids with a 3:1 ratio (96 and 32 molecules, respectively). Lipid bilayers were placed at the center of a rectangular periodic box in aqueous solutions consisting of 70 and 18 PolyZI molecules (DP = 2 and 8, respectively) with 13,200 waters and 32 potassium ions. The MD systems were optimized and heated gradually from −273 to 37 °C at 0.1 °C/ps. Production MD simulations were then performed at constant temperature (37 °C) and pressure (1 bar) for 500 ns. All bonds involving hydrogen atoms were constrained using the SHAKE algorithm with an integration time step of 2 fs and the non-bonded cut-off was set to 1 nm. The lipid bilayer was described by the Lipid17 force field^34^, and polyZI molecules were modeled using the general AMBER force fiel^35^. All the simulations were performed using the PMEMD and PMEMD.CUDA program modules of the Amber 20 package^36^ with an NVIDIA Pascal GPU system. The molecular graphics software PyMOL 2.5.0 (Schrödinger, Inc.) was used to visually examine the MD structures.

### Creation of liposome extrusion method

A 5 mg/mL DPPC suspension (Avanti) dissolved in chloroform was concentrated by an evaporator to form a thin film of DPPC on the bottom of the flask. PBS (1 mL) was put into the flask, and the thin film of DPPC was dispersed in the PBS solution. The lipid suspension was passed through a 0.1 μm pore filter to form liposomes, using a liposome extruder (Avanti).

### DSC of liposome in polyZI solution

The liposome concentration was adjusted to 1 mg/mL using PBS or 10% (w/v) polyZI/PBS. The DSC (PEAQ-DSC, Malvern Panalytical) conditions were; a measurement temperature range of 20 °C–60 °C, a heating rate of 60 °C/hr, and a low feedback mode.

### Statistical analysis

The experimental data were tested by one-way ANOVA with Dunnett test. Experimental data with two or more factors were tested by two-way ANOVA with the Tukey test. GraphPad Prism9 used for these tests.

### Reporting summary

Further information on research design is available in the Nature Portfolio Reporting Summary linked to this article.

### Supplementary information

Supplementary Information

Reporting Summary

## Data Availability

All data are available from the authors upon reasonable request.
