# Supplementary material for: Cell-compatible isotonic freezing media enabled by thermo-responsive osmolyte-adsorption/exclusion polymer matrices
Source: Commun Chem. 2023 Nov 29;6:260. doi: 10.1038/s42004-023-01061-7 (PMC10687075; doi:10.1038/s42004-023-01061-7)
Supplement: Supplementary file 1 — Supplementary Information [file 42004_2023_1061_MOESM1_ESM.pdf]

Supporting information

Cell-compatible isotonic freezing media enabled by thermo-responsive osmolyte-adsorption/exclusion polymer matrices

Yui Kato, Yuya Matsuda, Takuya Uto, Daisuke Tanaka, Kojiro Ishibashi, Takeru Ishizaki, Akio Ohta, Akiko Kobayashi, Masaharu Hazawa, Richard W. Wong, Kazuaki Ninomiya, Kenji Takahashi, Eishu Hirata, Kosuke Kuroda

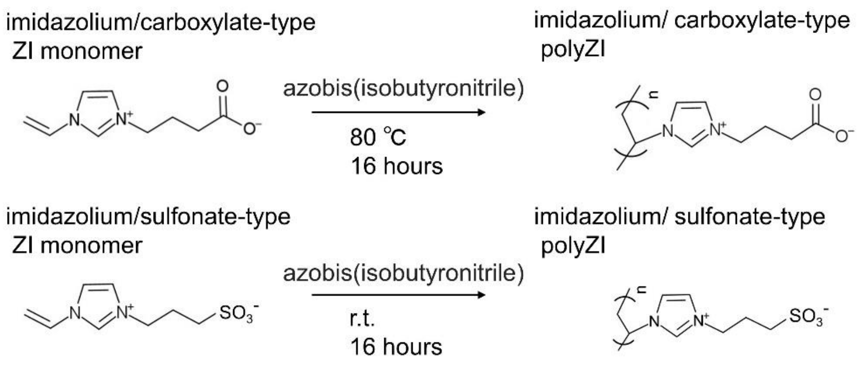

Supplementary Figure 1: Synthetic schemes of polyZIs.

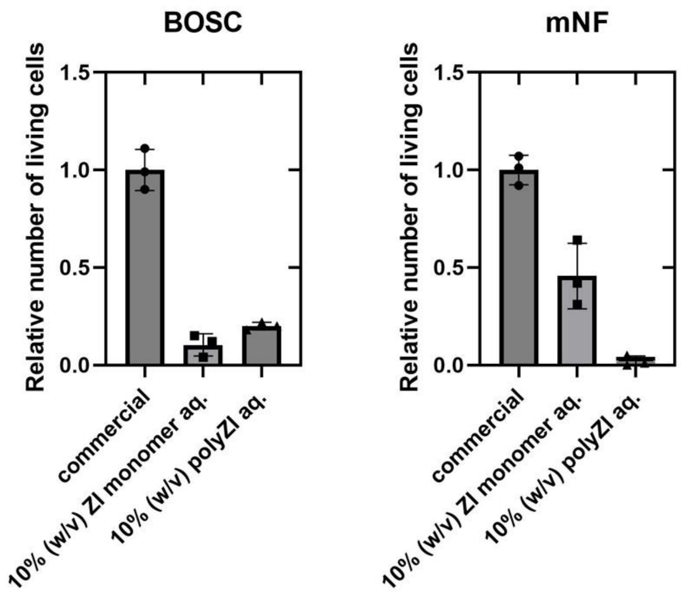

Supplementary Figure 2: Relative number of living BOSC cells and mNF after cryopreservation in the indicated solutions (n = 3, biological triplicates, one-way ANOVA). Commercial: Culture Sure freezing medium from Fujifilm Wako Pure Chemical Corporation. The statistical significances between commercial and the samples are p = 0.0002, 0.00021 (BOSC), p = 0.0069, 0.000029 (mNF).

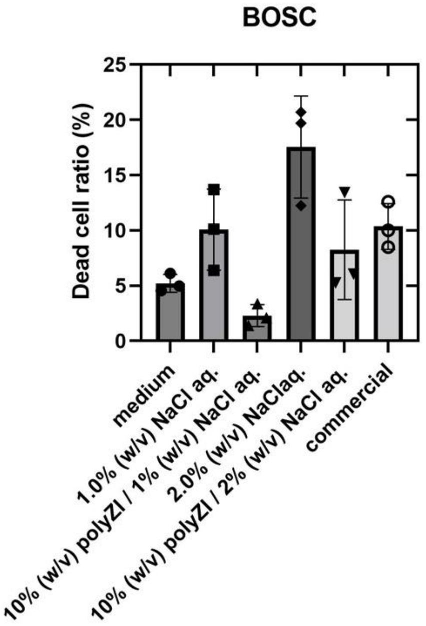

Supplementary Figure 3: Dead cell ratio of BOSC cells after a 60 min immersion in the indicated solutions (n = 3, biological triplicates, one-way ANOVA). These cells were incubated as floating cells in 10% polyZI solution after trypsinisation. Commercial: Culture Sure freezing medium from Fujifilm Wako Pure Chemical Corporation. The statistical significances between medium and the samples are p = 0.0088, 0.017, 0.31, 0.01,0.016.

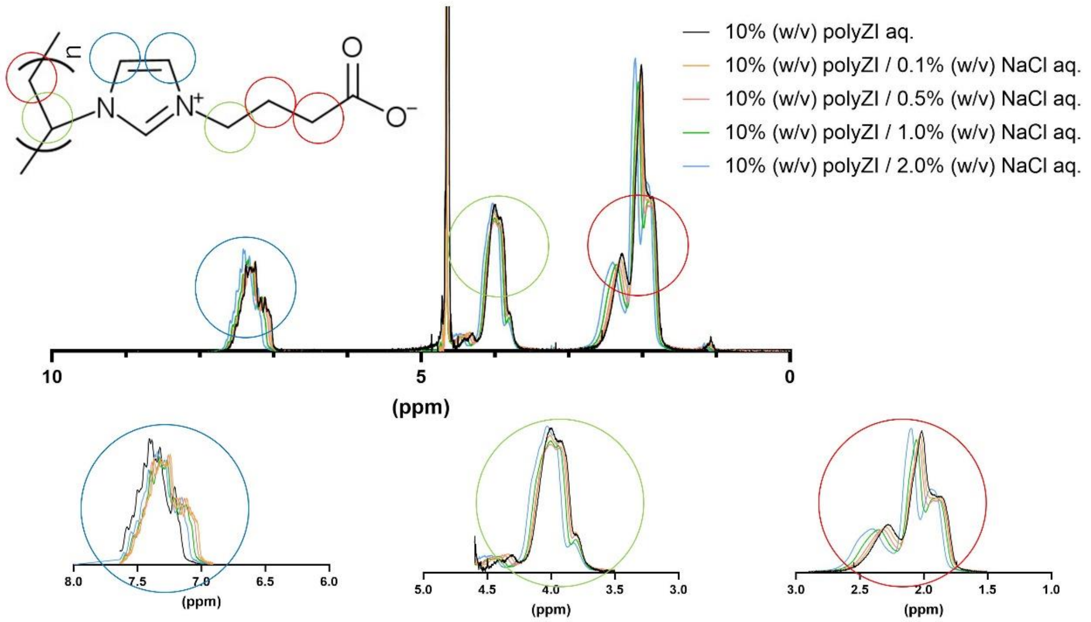

Supplementary Figure 4: ^{1}H nuclear magnetic resonance (NMR) spectra of the polyZI with 0–2% (w/v) NaCl. The solutions were prepared using deuterium oxide (D_2O).

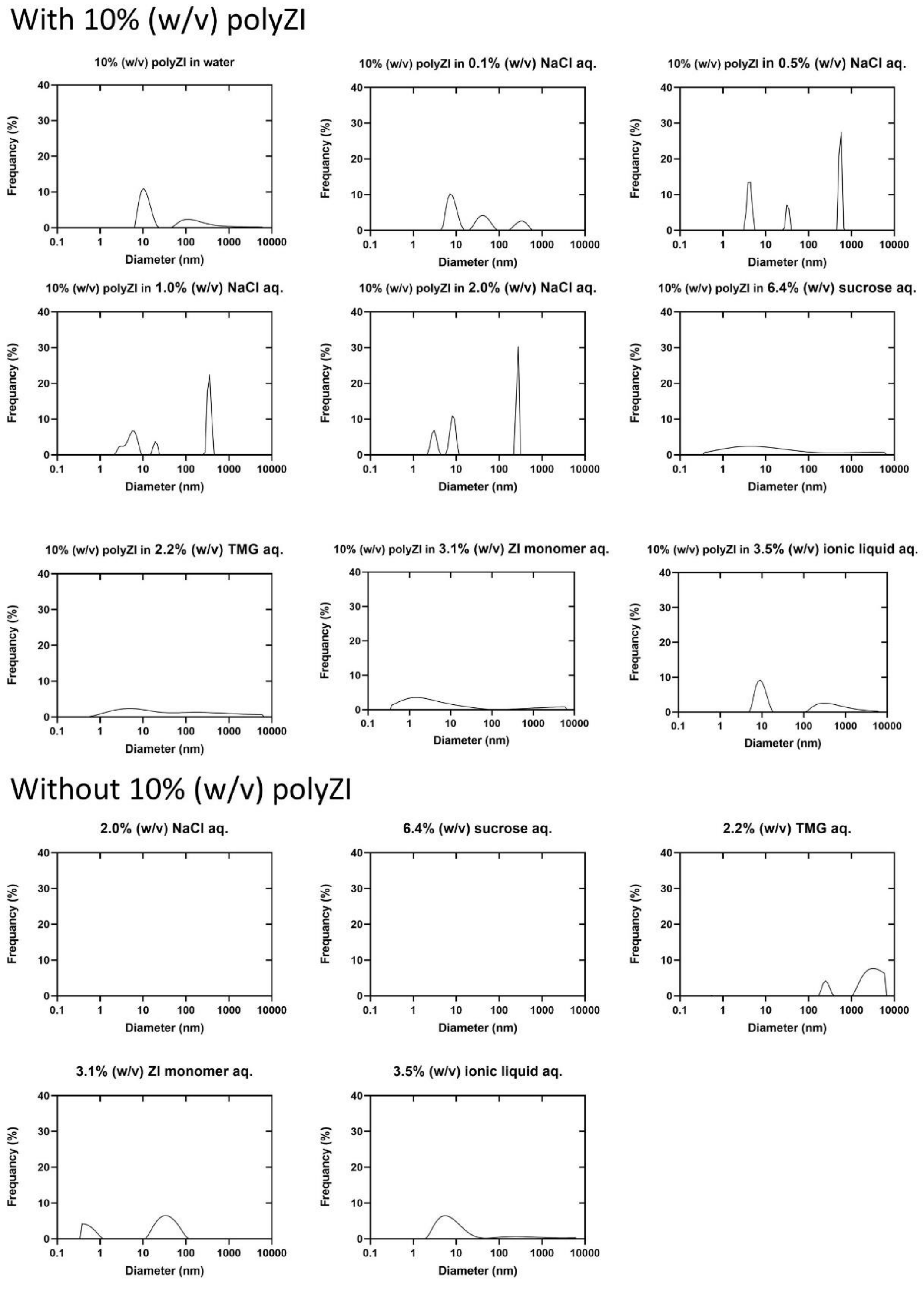

Supplementary Figure 5: PolyZI sizes observed via dynamic light scattering (DLS) in the polyZI solutions. The results of DLS were variable and the representative charts are shown.

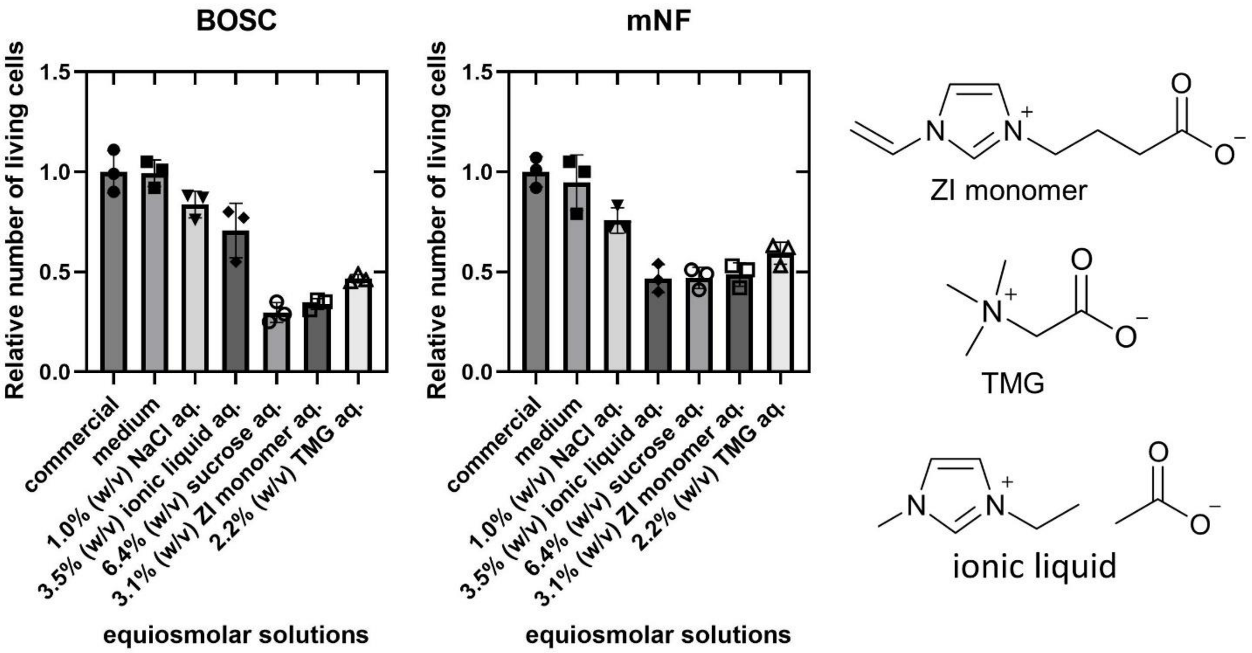

Supplementary Figure 6: The relative number of living BOSC cells and mNF after cryopreservation using 10% (w/v) polyZI solutions supplemented with the indicated solutes at the concentrations equimolar to 1% (w/v) NaCl ($n = 3$, biological triplicates, one-way ANOVA). Commercial: Culture Sure freezing medium from Fujifilm Wako Pure Chemical Corporation.

There are no statistical significance between the samples ($p > 0.1$).

Although the proportion of unfrozen water in the polyZI/sucrose aq. was 8% (Supplementary Table 1), the relative number of living cells after cryopreservation was low.

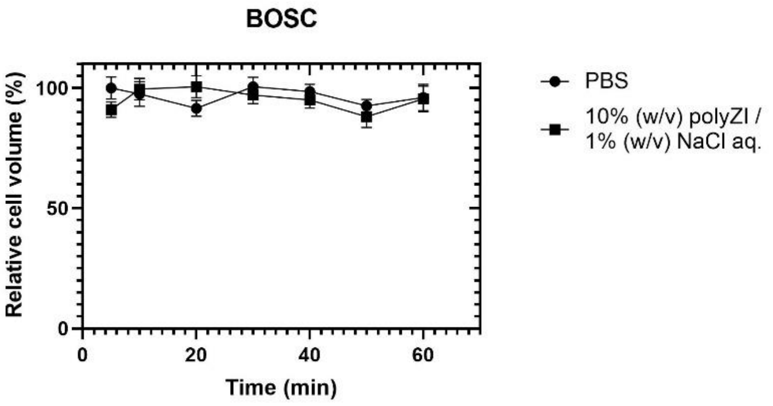

Supplementary Figure 7: Relative BOSC cell volume in 10% (w/v) polyZI / 10% (w/v) NaCl aq. and PBS (standardized as 100%) at room temperature (n = 3, biological triplicates). These cells were incubated as floating cells in the solutions after trypsinisation.

We sought to confirm the cell volume in the polyZI/NaCl aq. and found that it was equivalent to that in PBS. Therefore, the inhibition of intercellular ice crystal formation was not the primary cryoprotective mechanism of polyZI.

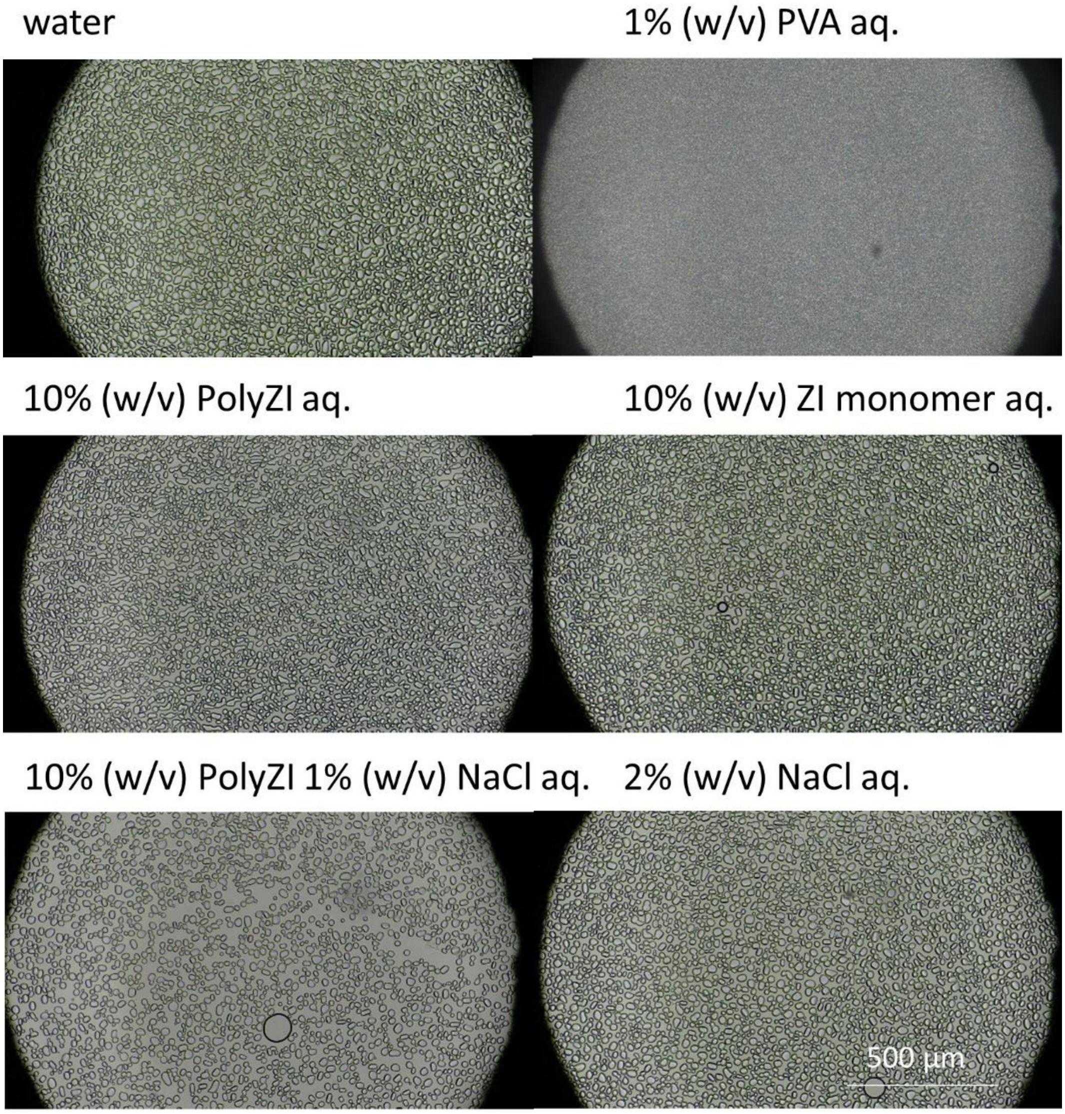

Supplementary Figure 8: Microscopic observation of ice formed in the indicated solutions.

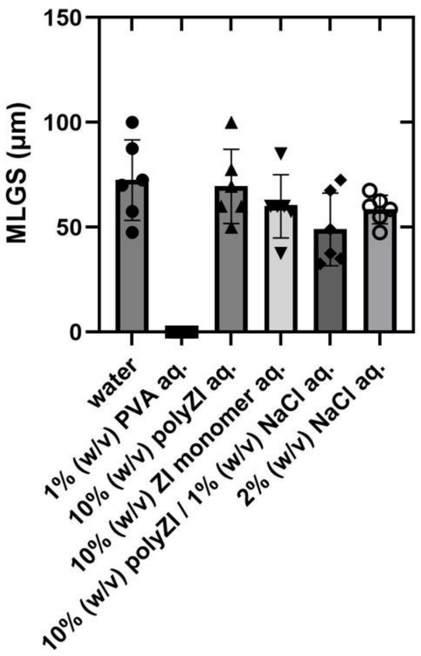

Supplementary Figure 9: MLGS in the indicated solutions. PVA: cannot be measured because the ice grain sizes are too small. There is no statistical significance between the samples except for 1% (w/v) PVA aq. ($p \geq 0.1$, one-way ANOVA).

The ice recrystallization activity was assayed via mean largest grain size (MLGS) that is the average size of the 5-10 largest crystals from each wafer.29 There was no significant difference.

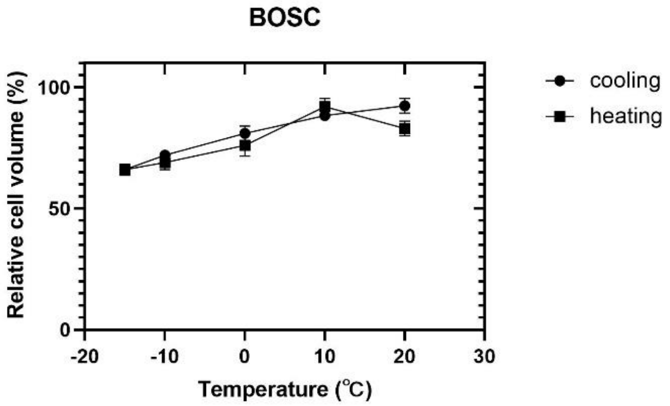

Supplementary Figure 10: Relative BOSC cell volume in 10% (w/v) polyZI / 1.0% (w/v) NaCl aq. during cooling and then heating at ±1 °C/min (n = 3, biological triplicates). These cells were incubated as floating cells after trypsinisation.

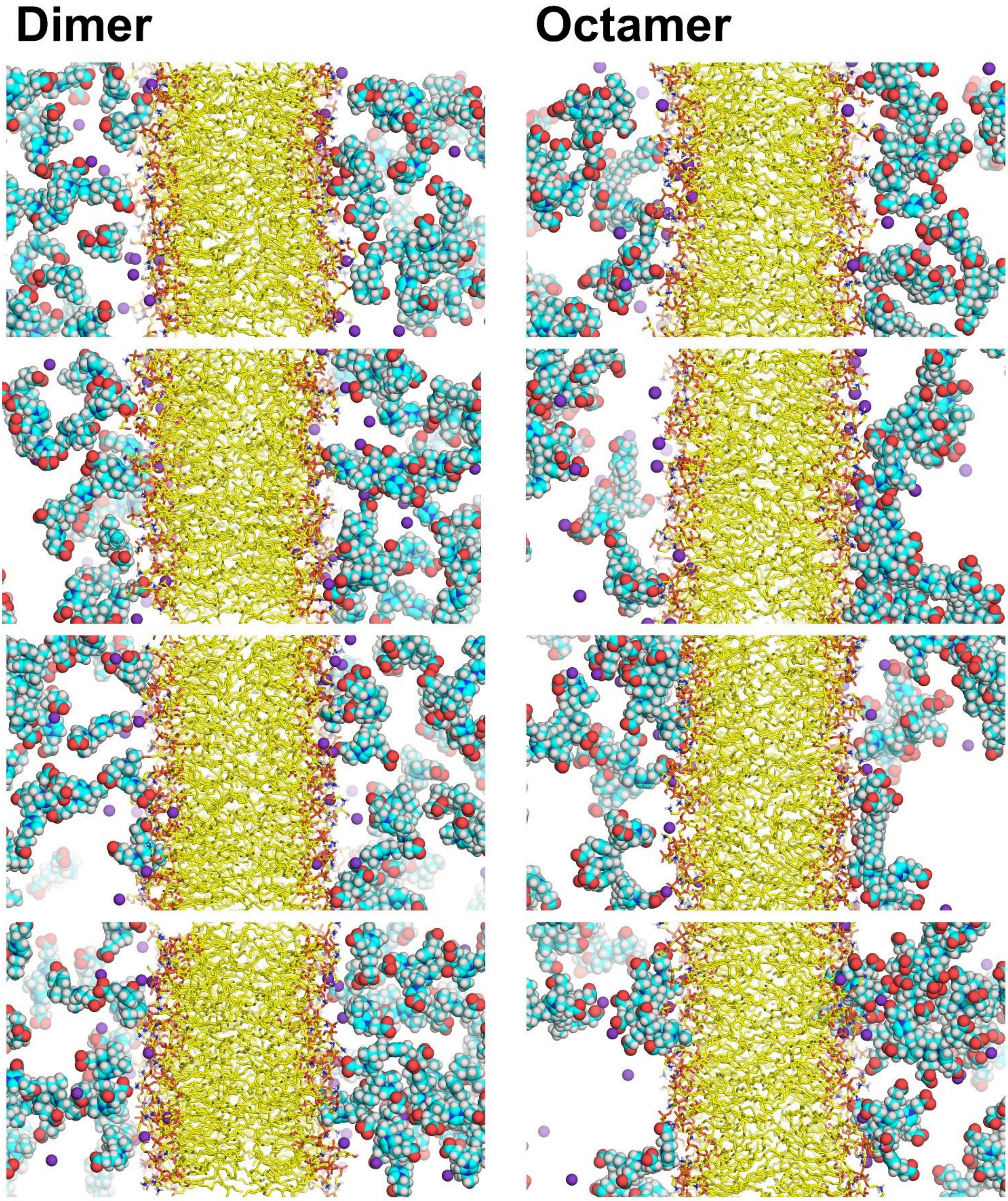

Supplementary Figure 11 Typical images of interaction between cell membrane and ZI dimer (left) and octamer (right) in the 10% (w/v) ZI oligomer aq.

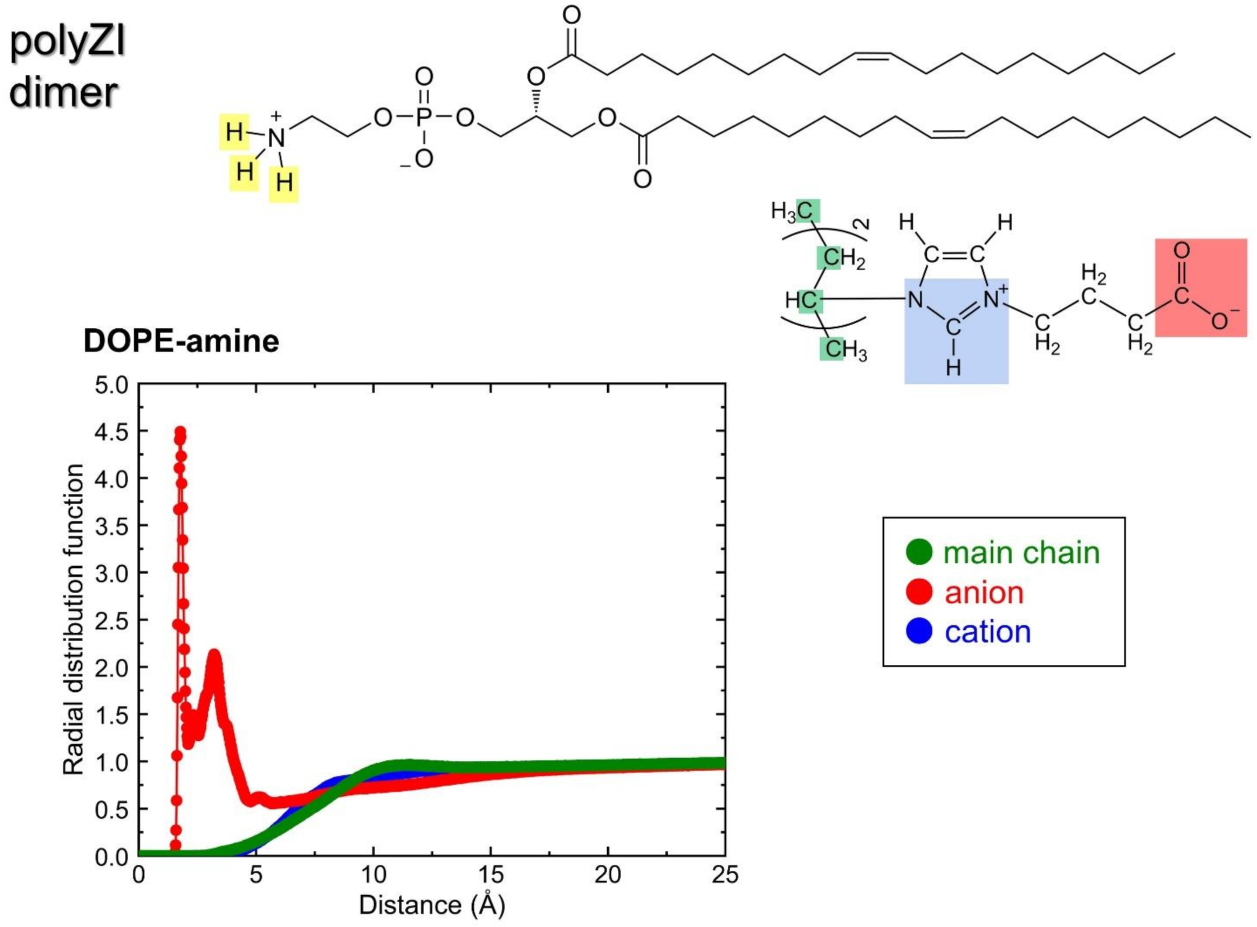

Supplementary Figure 12 Radial distribution functions involving the highlighted part of ZI dimer and DOPE-amino in 10% (w/v) ZI dimer aq. from MD trajectories for 1 μs.

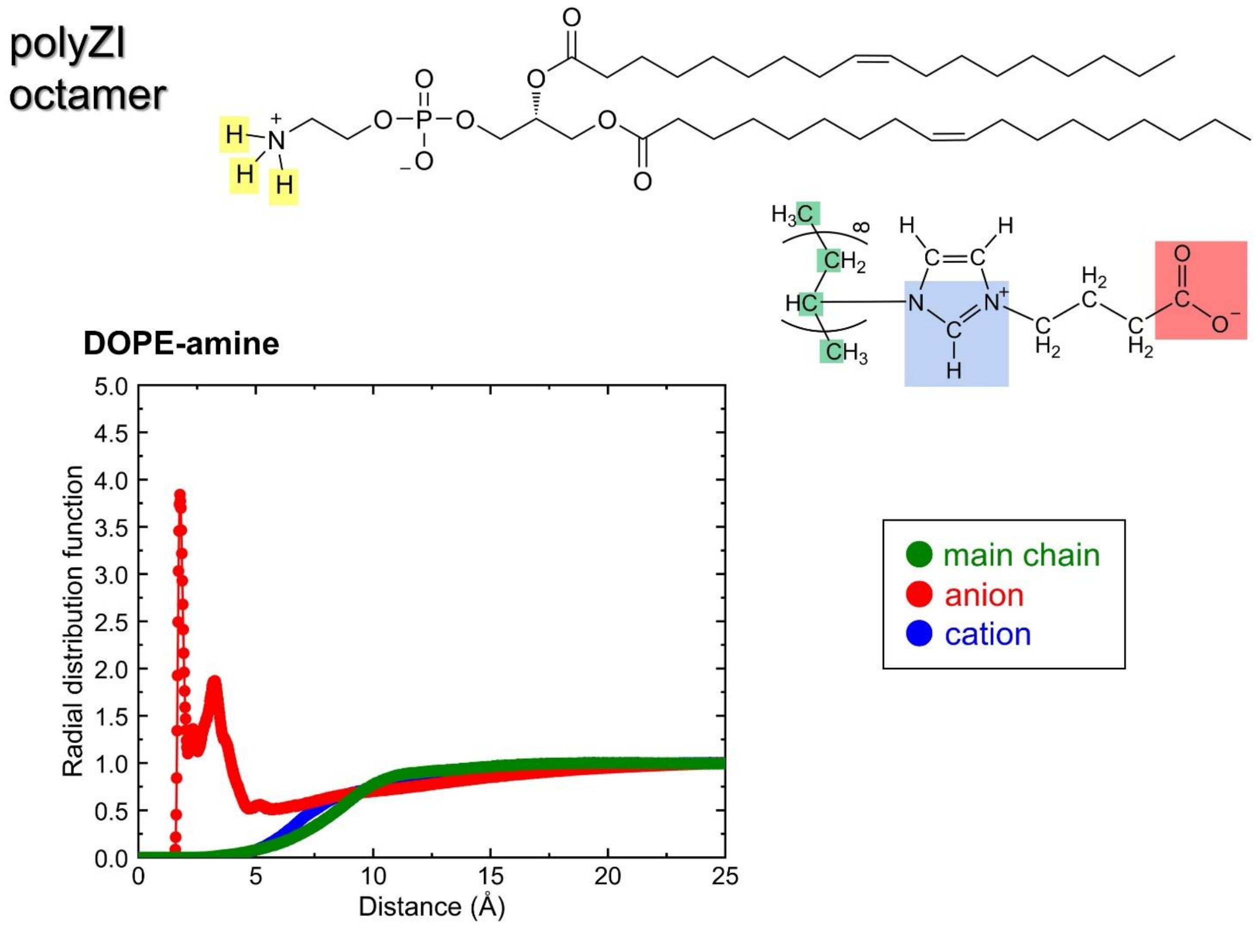

Supplementary Figure 13 Radial distribution functions involving the highlighted part of ZI octamer and DOPE-amino in 10% (w/v) ZI octamer aq. from MD trajectories for 1 μs.

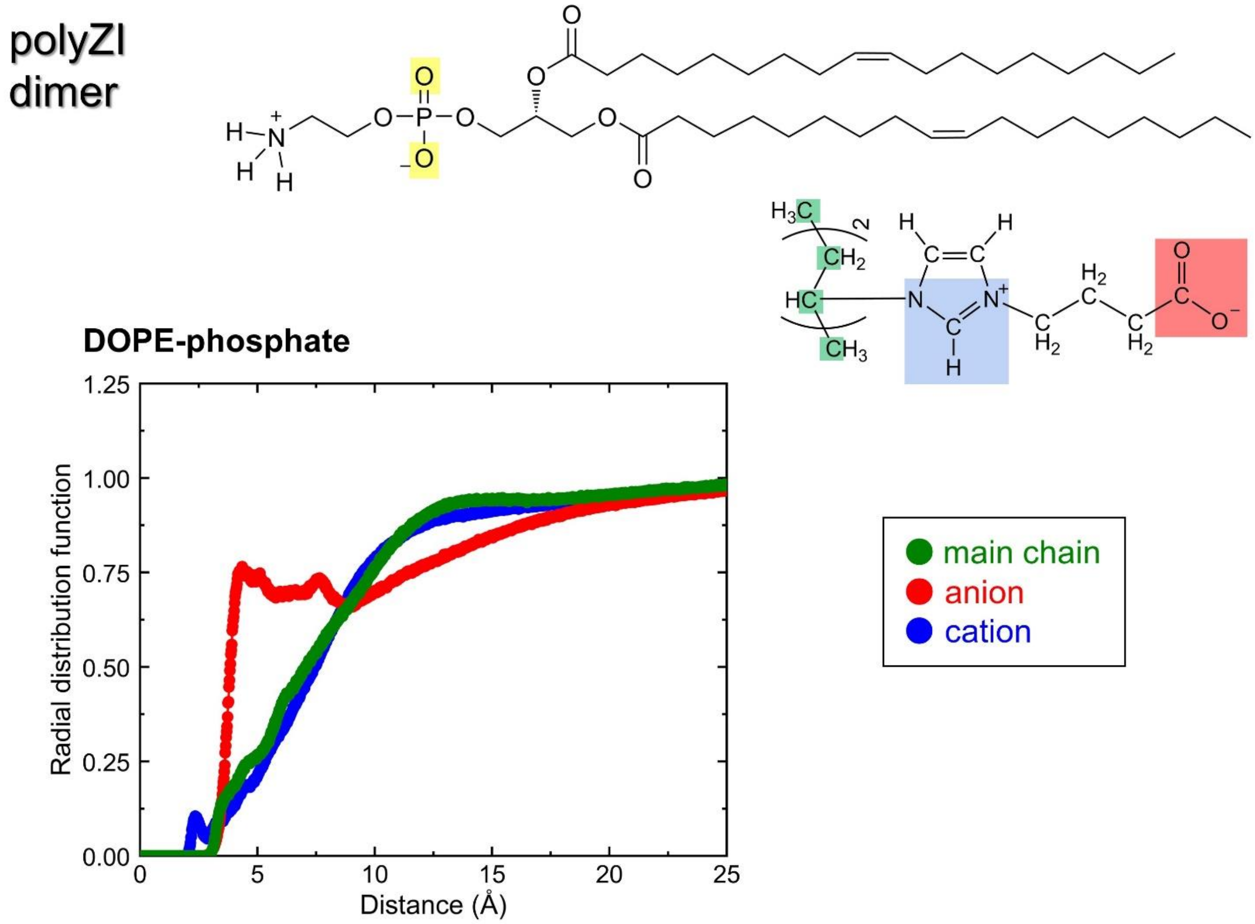

Supplementary Figure 14 Radial distribution functions involving the highlighted part of ZI dimer and DOPE-phosphate in 10% (w/v) ZI dimer aq. from MD trajectories for 1 μs.

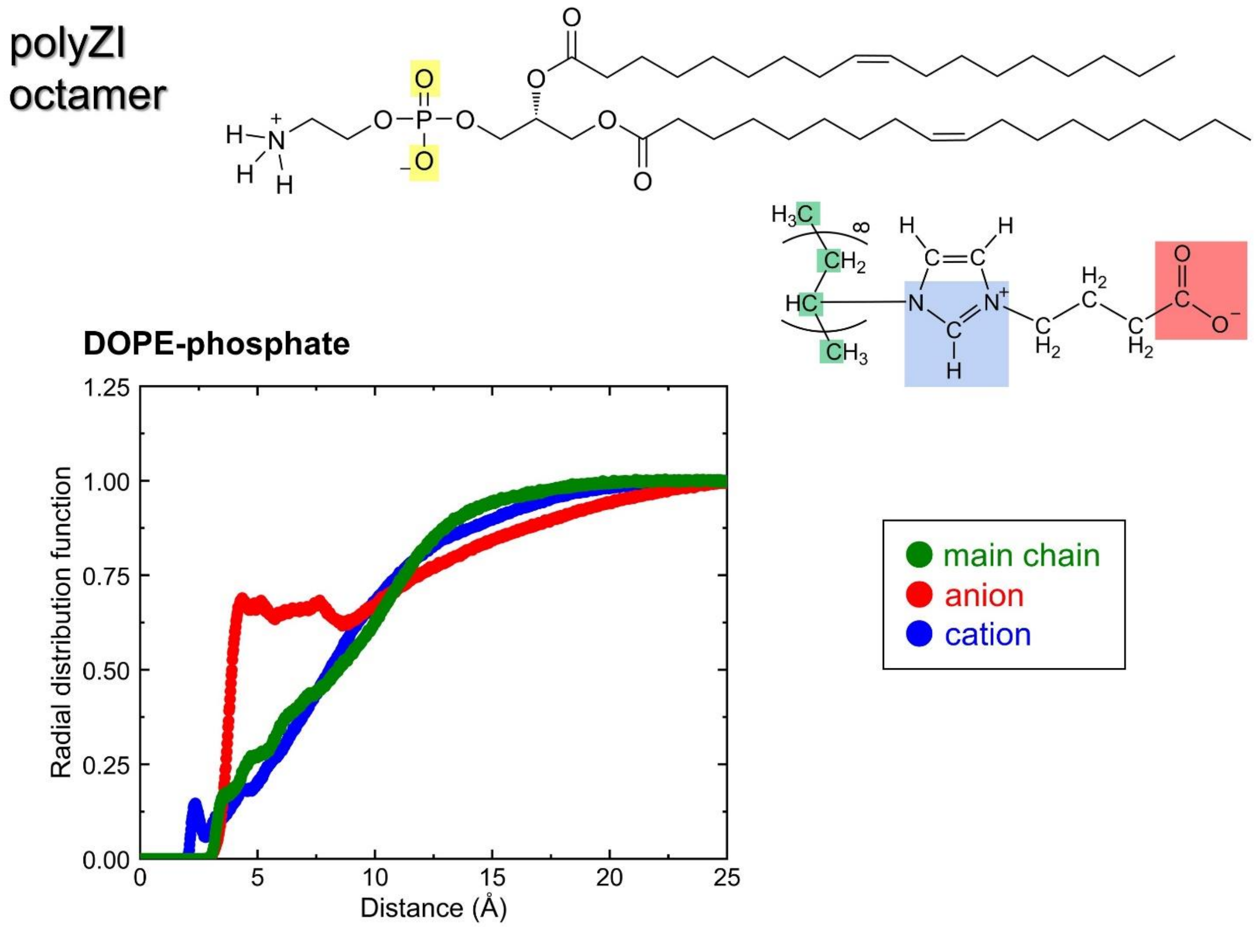

Supplementary Figure 15 Radial distribution functions involving the highlighted part of ZI octamer and DOPE-phosphate in 10% (w/v) ZI octamer aq. from MD trajectories for 1 μs.

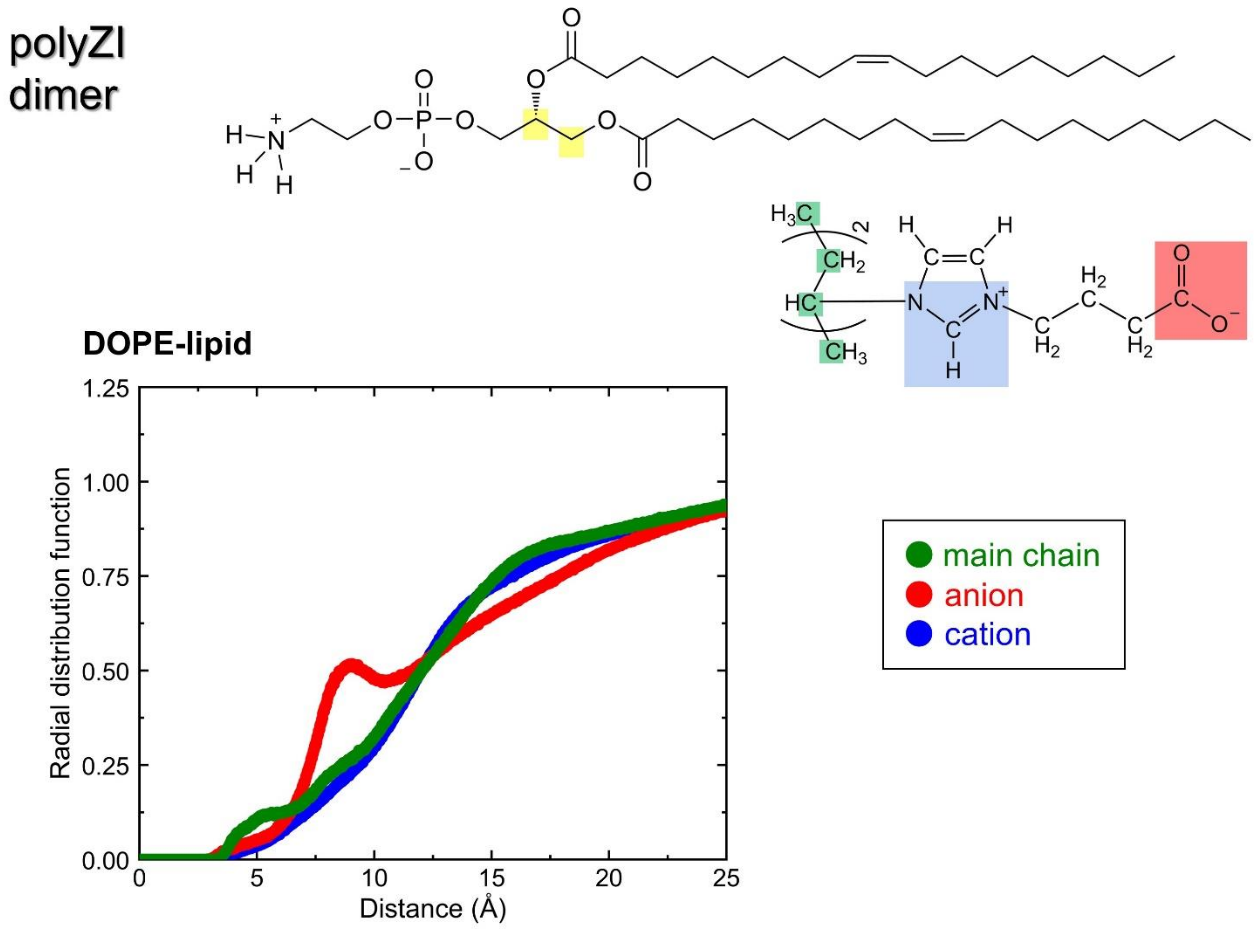

Supplementary Figure 16 Radial distribution functions involving the highlighted part of ZI dimer and DOPE-lipid in 10% (w/v) ZI dimer aq. from MD trajectories for 1 μs.

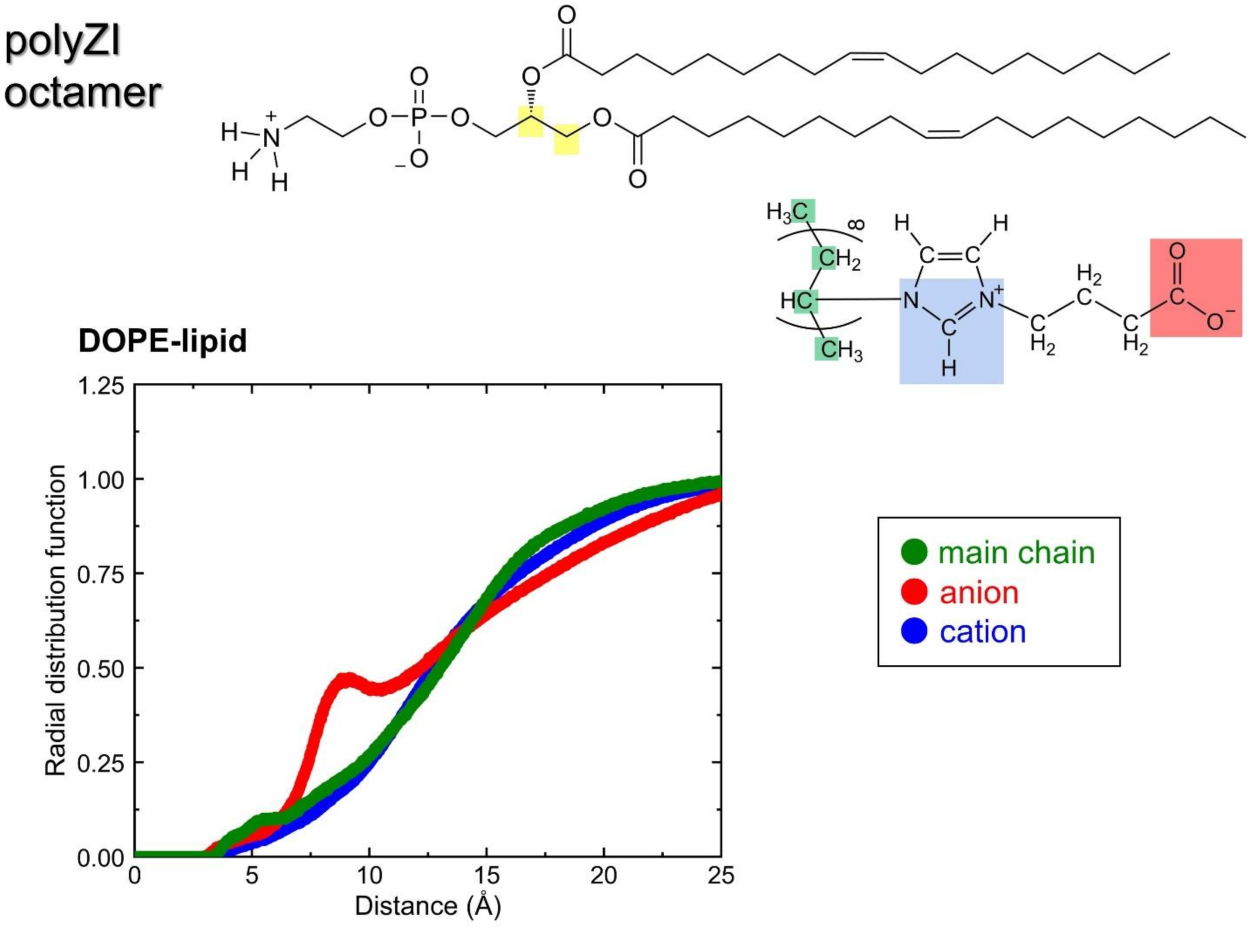

Supplementary Figure 17 Radial distribution functions involving the highlighted part of ZI dimer and DOPE-lipid in 10% (w/v) ZI dimer aq. from MD trajectories for 1 μs.

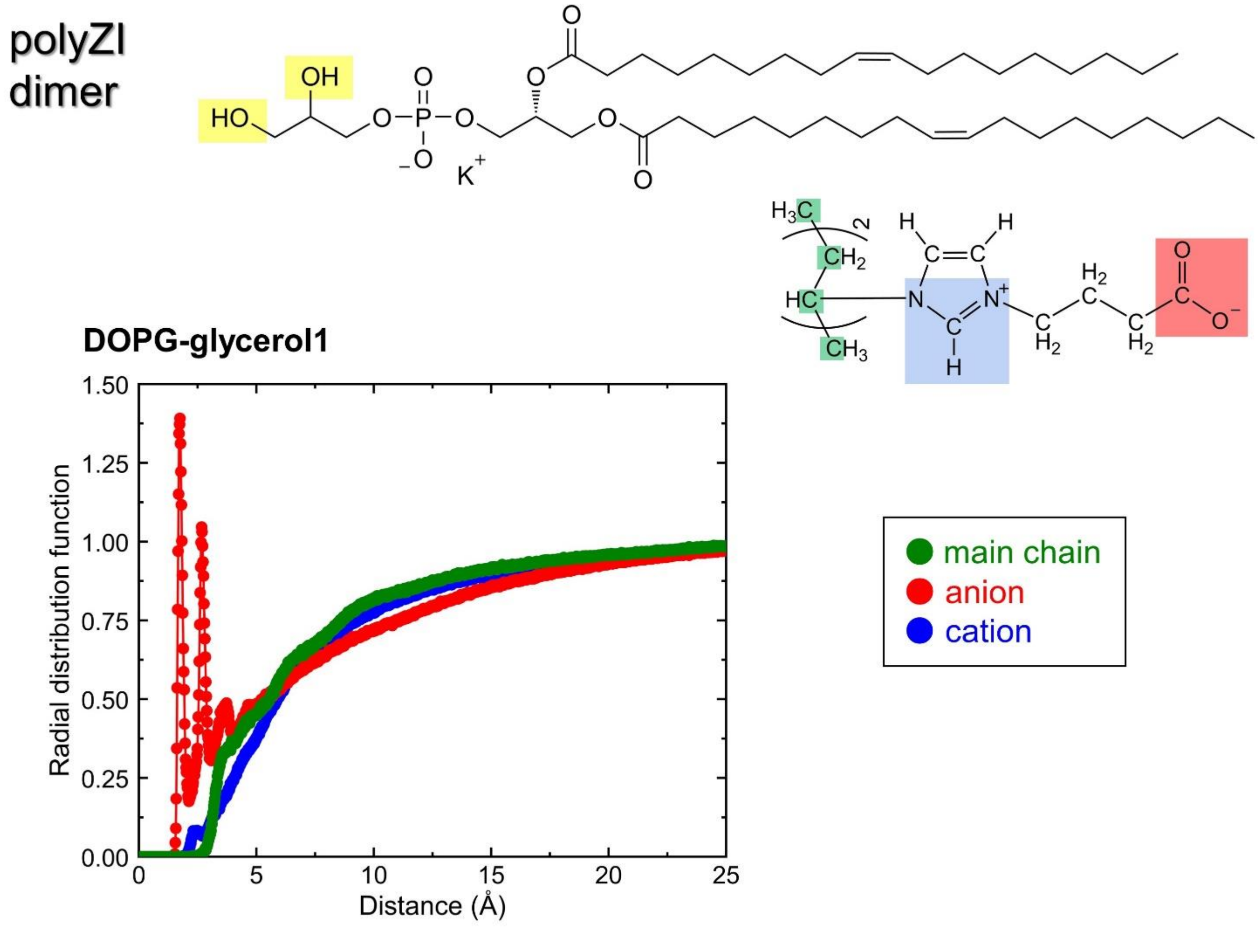

Supplementary Figure 18 Radial distribution functions involving the highlighted part of ZI dimer and DOPG-glycerol in 10% (w/v) ZI dimer aq. from MD trajectories for 1 μs.

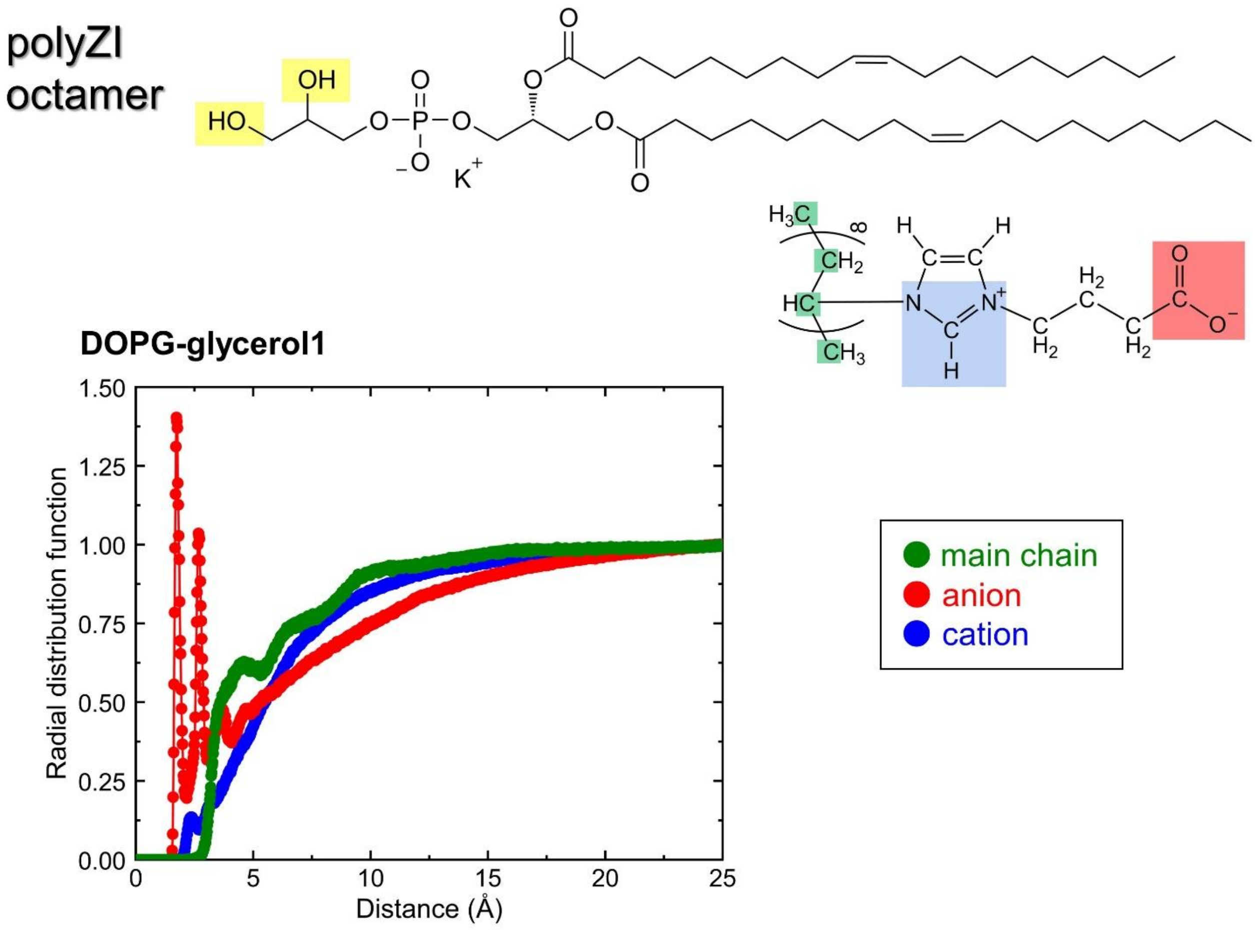

Supplementary Figure 19 Radial distribution functions involving the highlighted part of ZI octamer and DOPG-glycerol in 10% (w/v) ZI octamer aq. from MD trajectories for 1 μs.

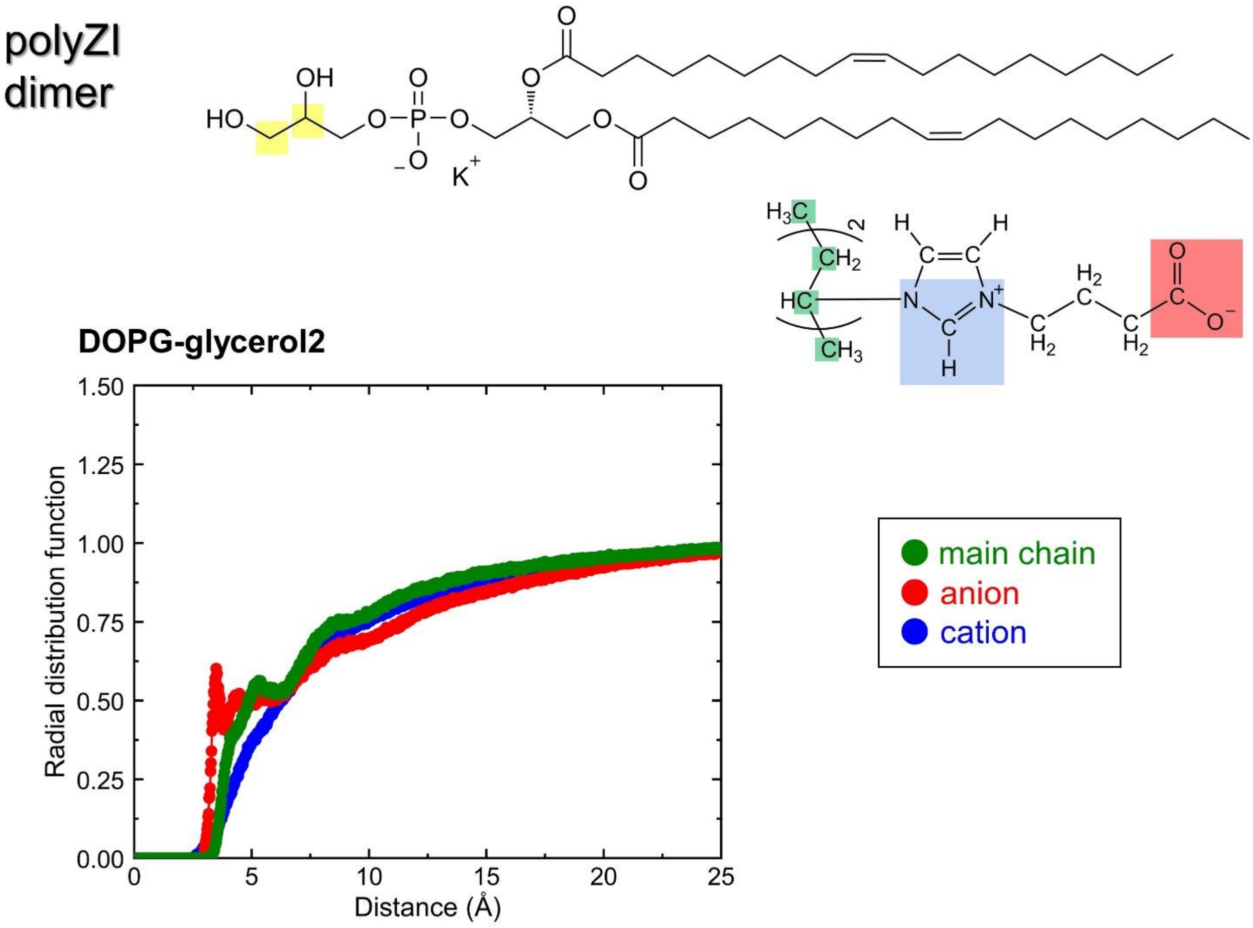

Supplementary Figure 20 Radial distribution functions involving the highlighted part of ZI dimer and DOPG-glycerol in 10% (w/v) ZI dimer aq. from MD trajectories for 1 μs.

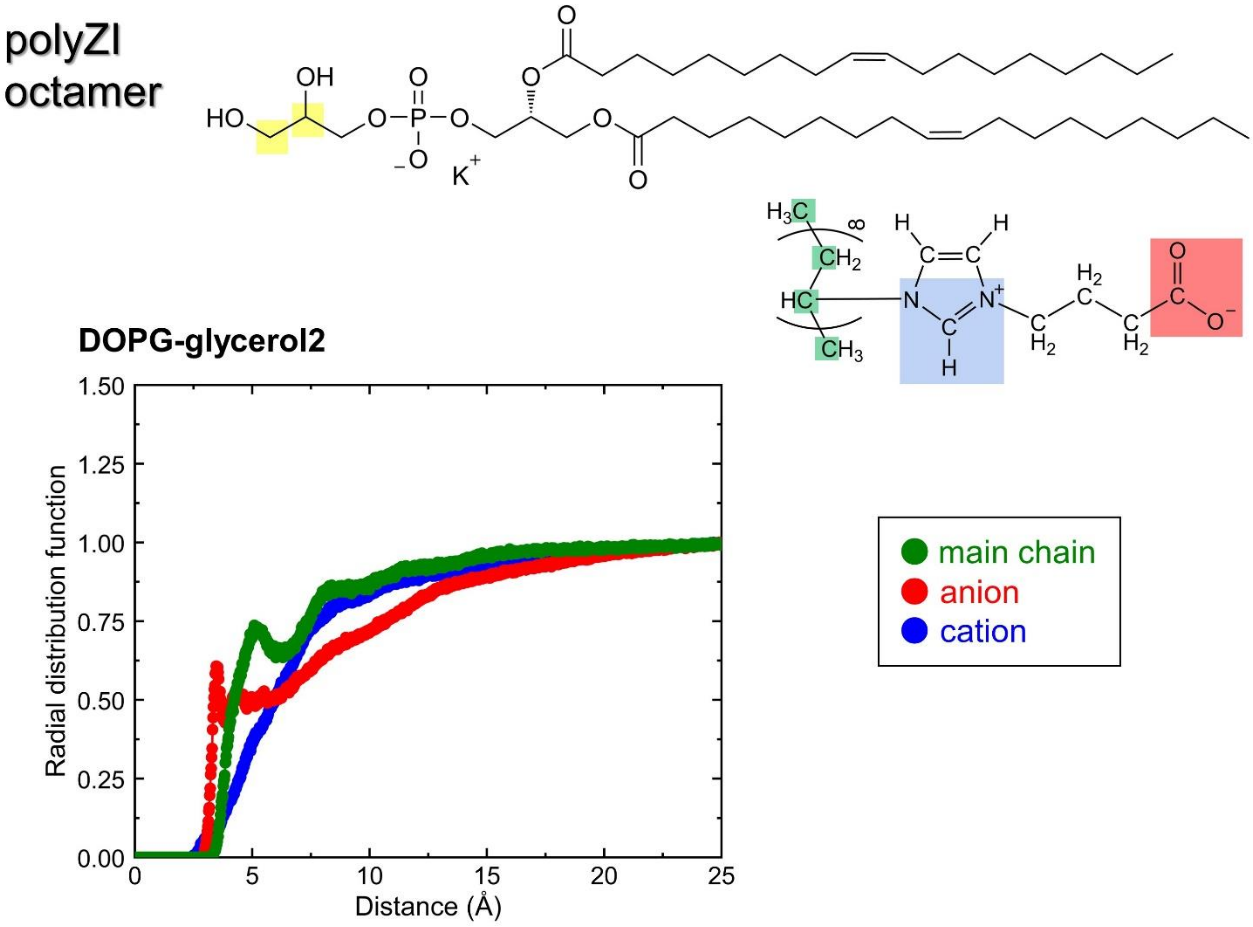

Supplementary Figure 21 Radial distribution functions involving the highlighted part of ZI octamer and DOPG-glycerol in 10% (w/v) ZI octamer aq. from MD trajectories for 1 μs.

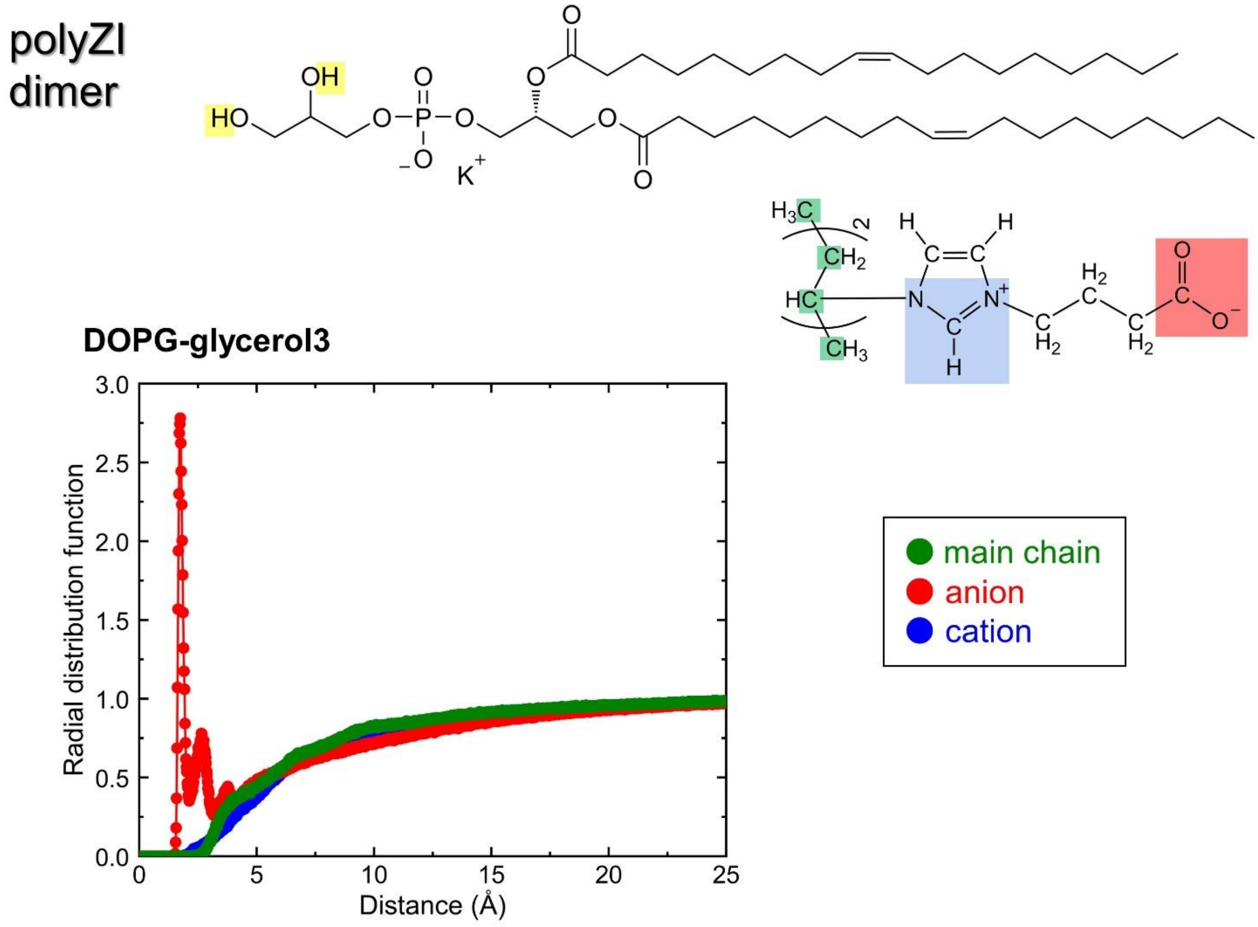

Supplementary Figure 22 Radial distribution functions involving the highlighted part of ZI dimer and DOPG-glycerol3 in 10% (w/v) ZI dimer aq. from MD trajectories for 1 μs.

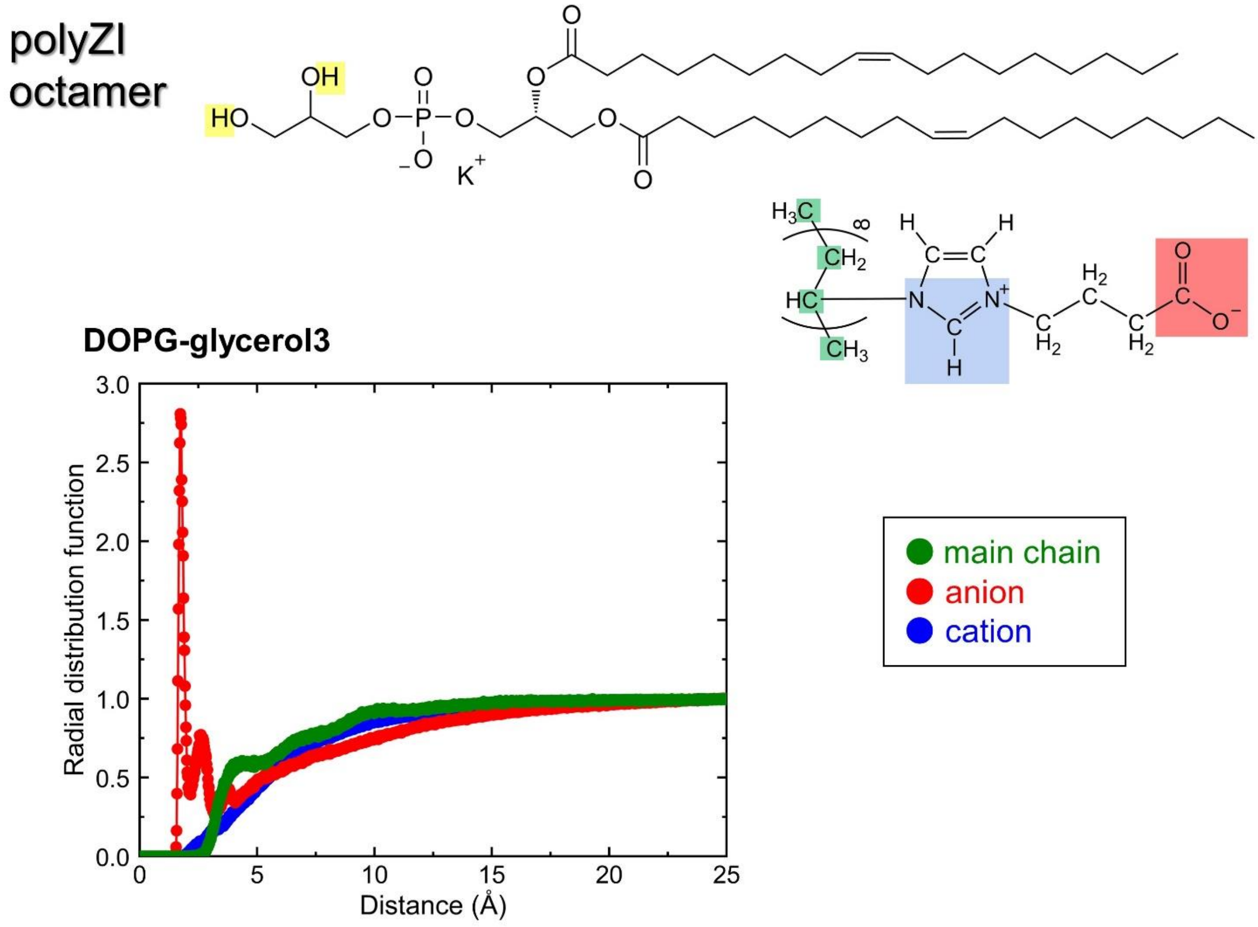

Supplementary Figure 23 Radial distribution functions involving the highlighted part of ZI octamer and DOPG-glycerol3 in 10% (w/v) ZI octamer aq. from MD trajectories for 1 μs.

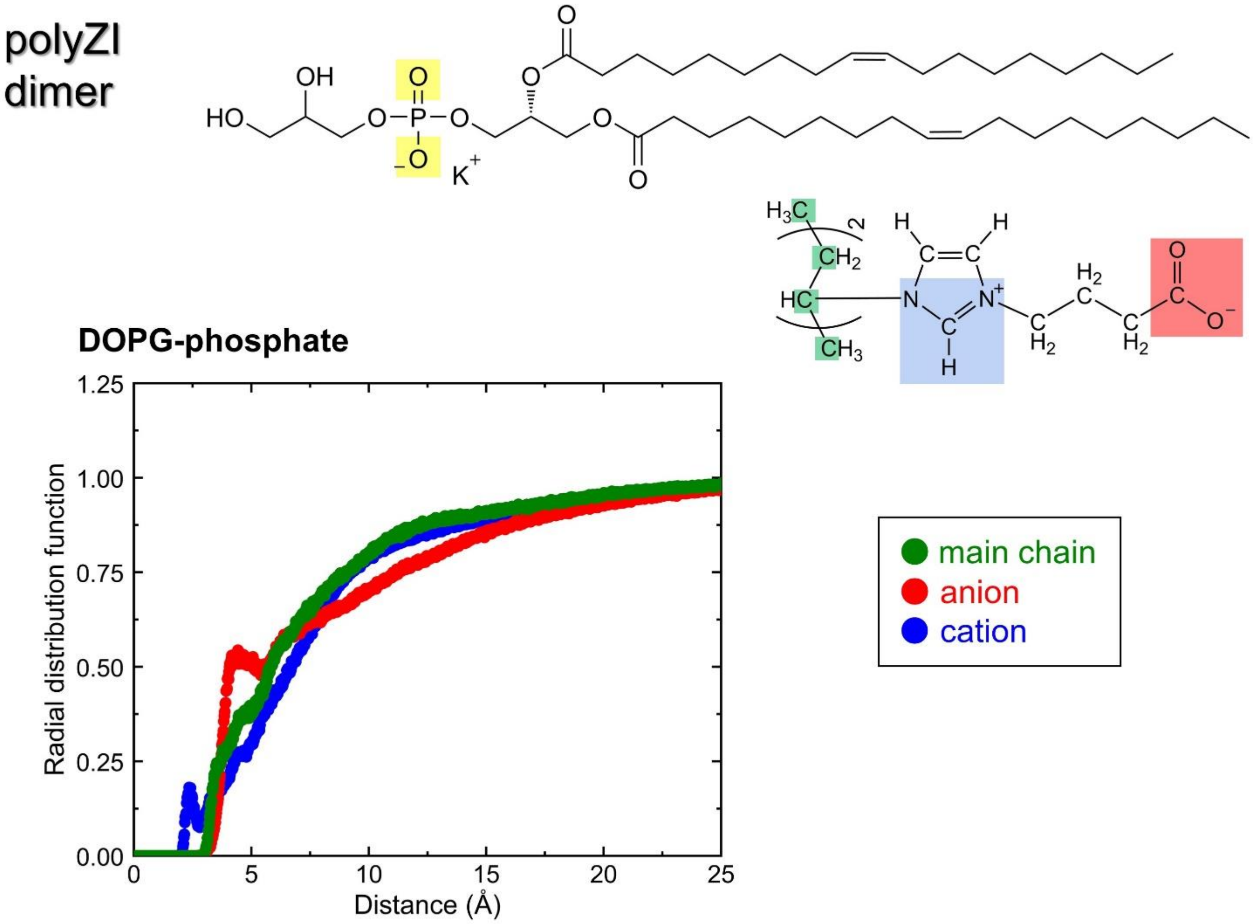

Supplementary Figure 24 Radial distribution functions involving the highlighted part of ZI dimer and DOPG-phosphate in 10% (w/v) ZI dimer aq. from MD trajectories for 1 μs.

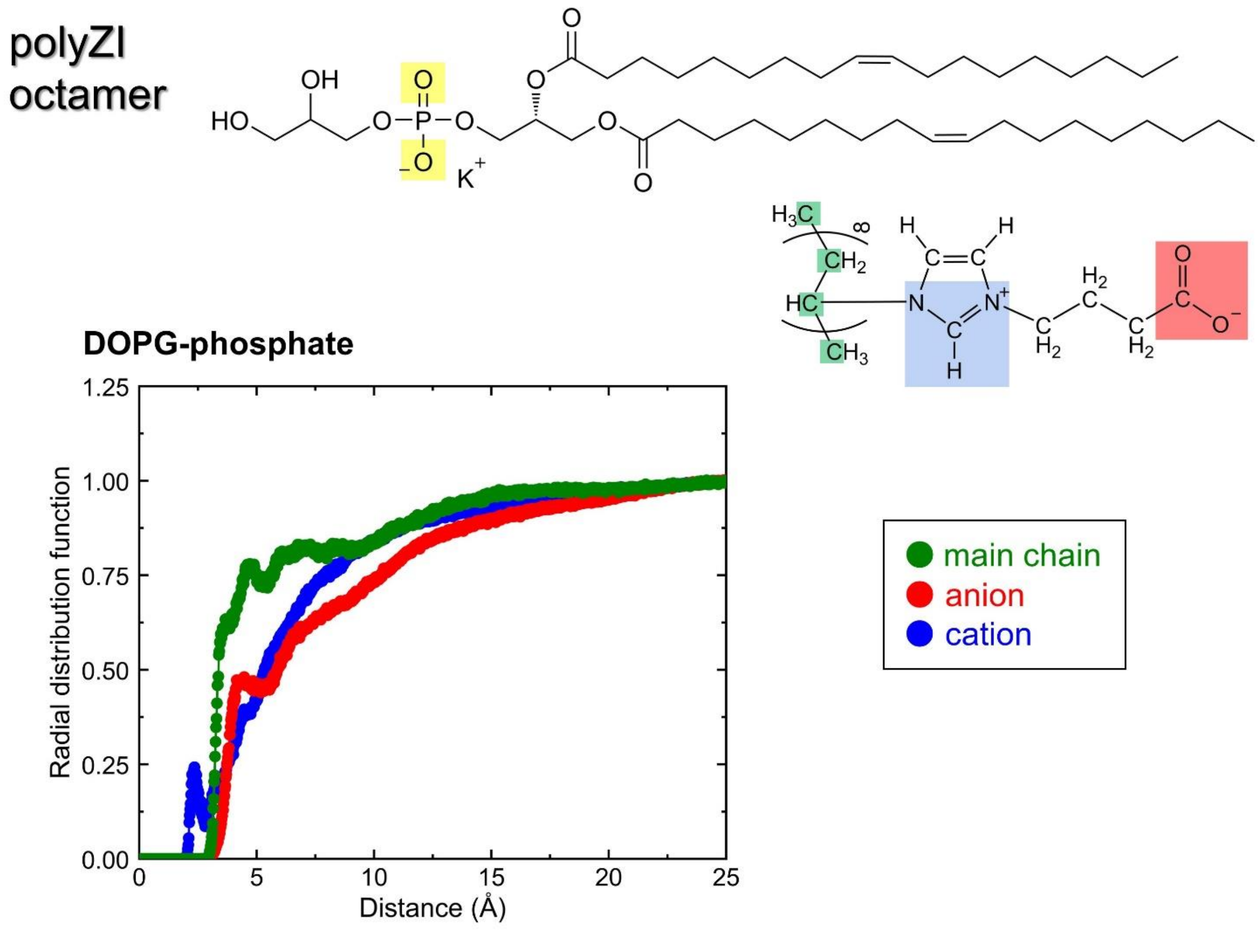

Supplementary Figure 25 Radial distribution functions involving the highlighted part of ZI octamer and DOPG-phosphate in 10% (w/v) ZI octamer aq. from MD trajectories for 1 μs.

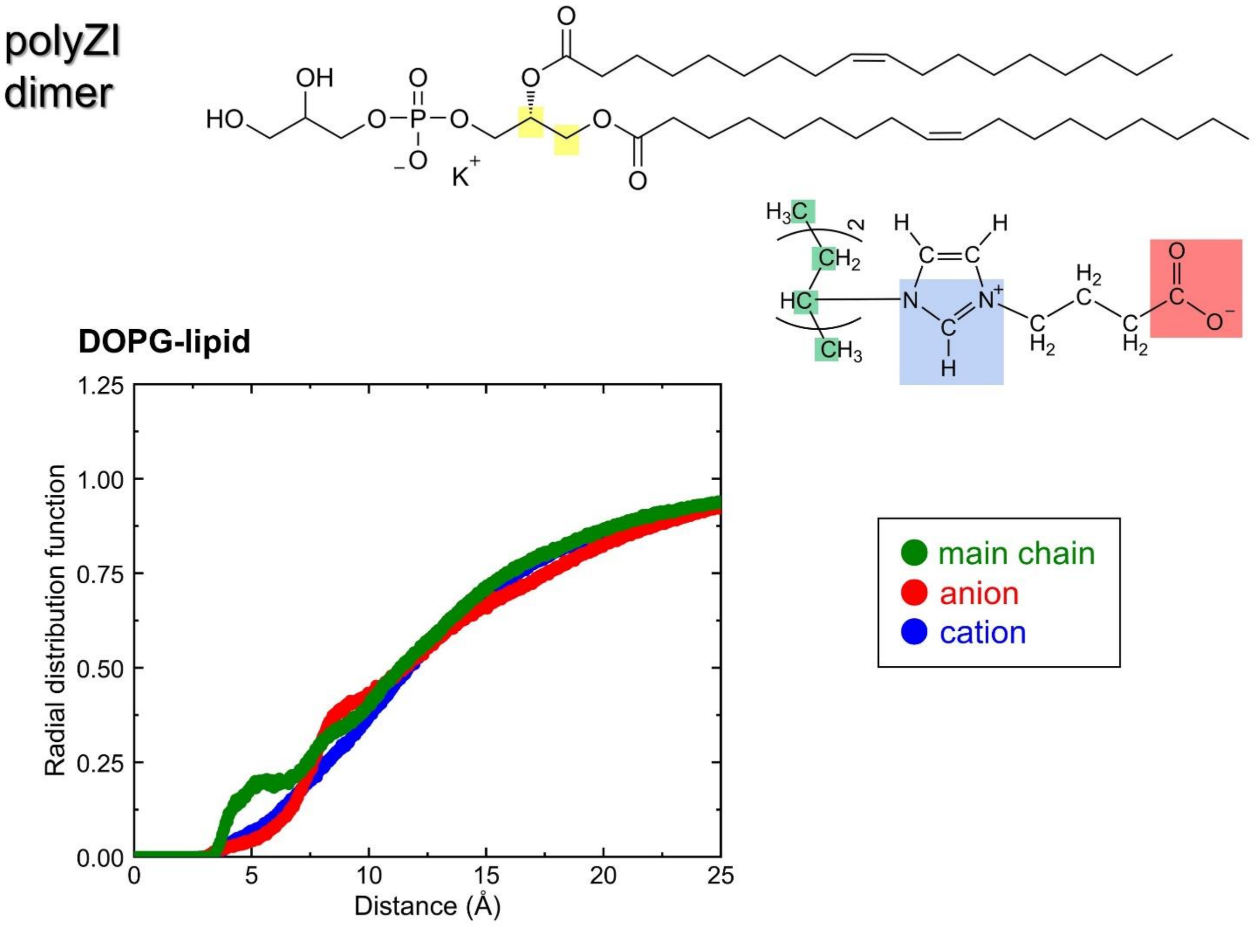

Supplementary Figure 26 Radial distribution functions involving the highlighted part of ZI dimer and DOPG-lipid in 10% (w/v) ZI dimer aq. from MD trajectories for 1 μs.

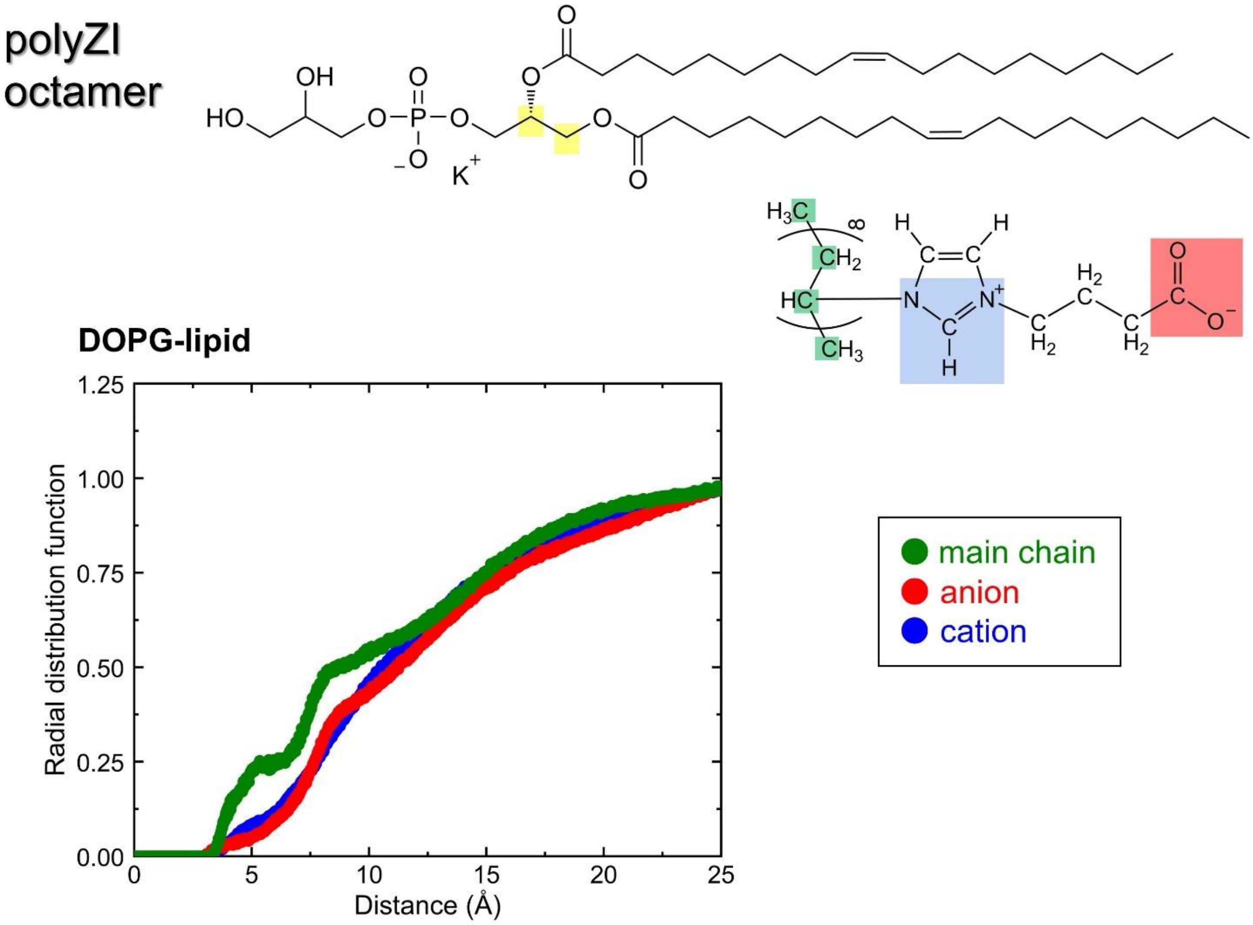

Supplementary Figure 27 Radial distribution functions involving the highlighted part of ZI octamer and DOPG-lipid in 10% (w/v) ZI octamer aq. from MD trajectories for 1 μs.

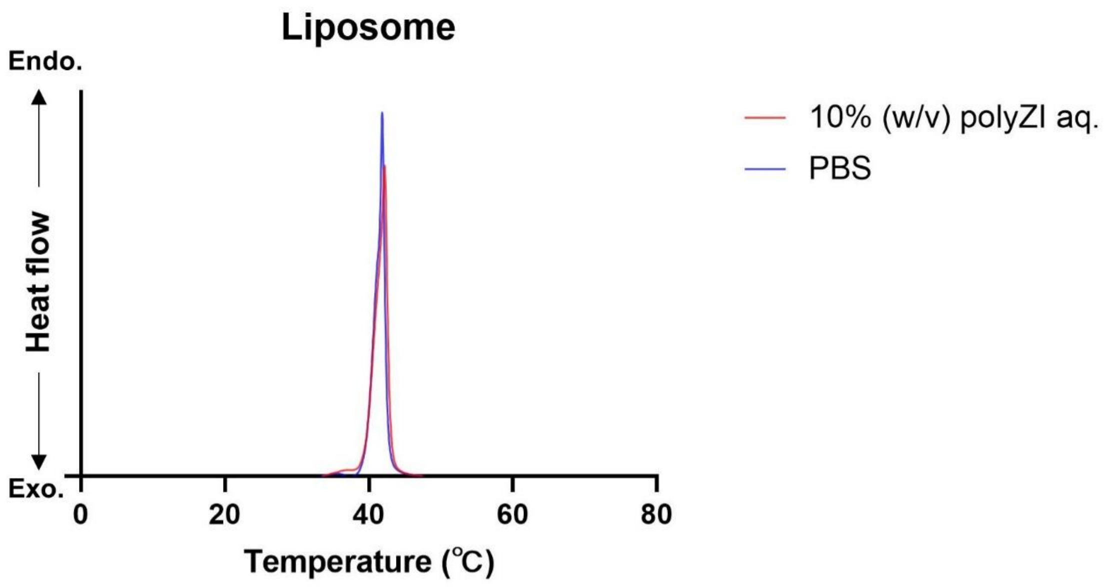

Supplementary Figure 28 DSC chart of liposomes in PBS and 10% (w/v) polyZI solutions.

Supplementary Table 1: Osmotic pressure and proportion of unfrozen water in the indicated solutions. The culture medium used in this study is Dulbecco's modified Eagle's medium (DMEM) with high glucose, and aq. indicates an aqueous solution comprising ultrapure water without any supplementation, unless noted.

	Osmotic pressure (mOsm)	Proportion of unfrozen water (%)
10% (w/v) polyZI aq.	5	0
10% (w/v) polyZI in culture medium	272	9
Culture medium	328	10
10% (w/v) polyZI + 1.0% (w/v) NaCl aq.	289	4
1% (w/v) NaCl aq.	327	11
10% (w/v) polyZI + 6.4% (w/v) sucrose aq.	157	8
6.4% (w/v) sucrose aq.	195	11
10% (w/v) polyZI + 3.1% (w/v) ZI monomer aq.	165	7
3.1% (w/v) ZI monomer aq.	230	8
10% (w/v) polyZI + 2.2% (w/v) TMG aq.	177	7
2.2% (w/v) TMG aq.	140	9
10% (w/v) polyZI + 3.5% (w/v) ionic liquid aq.	253	14
3.5% (w/v) ionic liquid aq.	386	-
10% (w/v) polyZI in PBS	254	4
PBS	280	-

Supplementary Table 2: Osmotic pressure of solutions and relative cell viability of human kidney cells (BOSC) and mouse normal fibroblasts (mNF) after cryopreservation using the indicated solutions. DMEM with high glucose was used as the medium in this study, and aq. indicates an aqueous solution comprising ultrapure water without any supplementation, unless noted.

	Osmotic pressure (mOsm)	Relative number of living cells (BOSC)	Relative number of living cells (mNF)
1% (w/v) NaCl aq.	327	-	-
10% (w/v) polyZI aq.	5	0.07	0.02
10% (w/v) polyZI 1.0% (w/v) NaCl aq.	289	0.84	0.76
10% (w/v) polyvinyl alcohol aq.	76	0.009	0.03
10% (w/v) polyvinyl alcohol 1.0% (w/v) NaCl aq.	397	0.02	0.03
10% (w/v) sodium polyacrylate aq.	59	0.02	0.03
10% (w/v) sodium polyacrylate 1% (w/v) NaCl aq.	354	0.07	0.08
