# Supplementary material for: Cell-compatible isotonic freezing media enabled by thermo-responsive osmolyte-adsorption/exclusion polymer matrices
Source: Commun Chem. 2023 Nov 29;6:260. doi: 10.1038/s42004-023-01061-7 (PMC10687075; doi:10.1038/s42004-023-01061-7)
Supplement: Supplementary file 2 — Reporting Summary [file 42004_2023_1061_MOESM2_ESM.pdf]

nature research

Corresponding author(s): Kosuke Kuroda, Eishu Hirata

Last updated by author(s): Oct 20, 2023

Reporting Summary

Nature Research wishes to improve the reproducibility of the work that we publish. This form provides structure for consistency and transparency in reporting. For further information on Nature Research policies, see our Editorial Policies and the Editorial Policy Checklist.

Statistics

For all statistical analyses, confirm that the following items are present in the figure legend, table legend, main text, or Methods section.

n/a	Confirmed	
☐	☒	The exact sample size (*n*) for each experimental group/condition, given as a discrete number and unit of measurement
☐	☒	A statement on whether measurements were taken from distinct samples or whether the same sample was measured repeatedly
☐	☒	The statistical test(s) used AND whether they are one- or two-sided<br>*Only common tests should be described solely by name; describe more complex techniques in the Methods section.*
☒	☐	A description of all covariates tested
☒	☐	A description of any assumptions or corrections, such as tests of normality and adjustment for multiple comparisons
☐	☒	A full description of the statistical parameters including central tendency (e.g. means) or other basic estimates (e.g. regression coefficient) AND variation (e.g. standard deviation) or associated estimates of uncertainty (e.g. confidence intervals)
☒	☐	For null hypothesis testing, the test statistic (e.g. *F*, *t*, *r*) with confidence intervals, effect sizes, degrees of freedom and *P* value noted<br>*Give P values as exact values whenever suitable.*
☒	☐	For Bayesian analysis, information on the choice of priors and Markov chain Monte Carlo settings
☒	☐	For hierarchical and complex designs, identification of the appropriate level for tests and full reporting of outcomes
☒	☐	Estimates of effect sizes (e.g. Cohen's *d*, Pearson's *r*), indicating how they were calculated

Our web collection on statistics for biologists contains articles on many of the points above.

Software and code

Policy information about availability of computer code

Data collection	Phase contrast images were acquired with Olympus IX83 using cellSens Dimension software and using a microscope (ECLIPSE Ts2, Nikon Corporation) and a camera (FLOYD, WRAYMER INC.) . The phase behaviour of the solutions was analysed by DSC-60A plus (Shimadzu corporation) and TA-60WS software (Shimadzu corporation). Number of cells were counted by hemocytometer (Fukaekasei Corporation and Watson Corporation) with trypan blue (Fujifilm Wako Pure Chemical Corporation). The images of the ice crystals were captured using a microscope (ECLIPSE Ts2, Nikon Corporation) with a cooling stage (10002L, JAPAN HIGH TECH CO.,LTD.). 1H NMR was measured to check the structure of polyZI using ECA400 (external magnetic field 400 MHz) manufactured by JEOL Ltd. For particle size distribution, a DLS device manufactured by HORIBA, Ltd. was used.
Data analysis	All the statistical data were analyzed with Microsoft Excel and/or GraphPad Prism 7.

For manuscripts utilizing custom algorithms or software that are central to the research but not yet described in published literature, software must be made available to editors and reviewers. We strongly encourage code deposition in a community repository (e.g. GitHub). See the Nature Research guidelines for submitting code & software for further information.

Data

Policy information about availability of data

All manuscripts must include a data availability statement. This statement should provide the following information, where applicable:

- Accession codes, unique identifiers, or web links for publicly available datasets
- A list of figures that have associated raw data
- A description of any restrictions on data availability

All data are available from the authors upon reasonable request.

Field-specific reporting

Please select the one below that is the best fit for your research. If you are not sure, read the appropriate sections before making your selection.

☒ Life sciences ☐ Behavioural & social sciences ☐ Ecological, evolutionary & environmental sciences

For a reference copy of the document with all sections, see nature.com/documents/nr-reporting-summary-flat.pdf

Life sciences study design

All studies must disclose on these points even when the disclosure is negative.

Sample size	Sample size was determined based on previous experience and published literature.
Data exclusions	No data were excluded from the study
Replication	Experiments were carried out with the number of replicates indicated in the manuscript. All attempts at replication were successful.
Randomization	Randomization was not necessary for the experiments.
Blinding	No blinding.

Reporting for specific materials, systems and methods

We require information from authors about some types of materials, experimental systems and methods used in many studies. Here, indicate whether each material, system or method listed is relevant to your study. If you are not sure if a list item applies to your research, read the appropriate section before selecting a response.

Materials & experimental systems

n/a	Involved in the study	
☒	☐	Antibodies
☒	☐	Eukaryotic cell lines
☒	☐	Palaeontology and archaeology
☒	☐	Animals and other organisms
☒	☐	Human research participants
☒	☐	Clinical data
☒	☐	Dual use research of concern

Methods

n/a	Involved in the study	
☒	☐	ChIP-seq
☒	☐	Flow cytometry
☒	☐	MRI-based neuroimaging
